# Representations and generalization in artificial and brain neural networks

**DOI:** 10.1073/pnas.2311805121

**Published:** 2024-06-24

**Authors:** Qianyi Li, Ben Sorscher, Haim Sompolinsky

**Affiliations:** ^a^The Harvard Biophysics Graduate Program, Harvard University, Cambridge, MA 02138; ^b^Center for Brain Science, Harvard University, Cambridge, MA 02138; ^c^The Applied Physics Department, Stanford University, Stanford, CA 94305; ^d^Edmond and Lily Safra Center for Brain Sciences, Hebrew University, Jerusalem 9190401, Israel

**Keywords:** deep neural networks, visual cortex, neural manifolds, few-shot learning, representational drift

## Abstract

Humans and animals excel at generalizing from limited data, a capability yet to be fully replicated in artificial intelligence. This perspective investigates generalization in biological and artificial deep neural networks (DNNs), in both in-distribution and out-of-distribution contexts. We introduce two hypotheses: First, the geometric properties of the neural manifolds associated with discrete cognitive entities, such as objects, words, and concepts, are powerful order parameters. They link the neural substrate to the generalization capabilities and provide a unified methodology bridging gaps between neuroscience, machine learning, and cognitive science. We overview recent progress in studying the geometry of neural manifolds, particularly in visual object recognition, and discuss theories connecting manifold dimension and radius to generalization capacity. Second, we suggest that the theory of learning in wide DNNs, especially in the thermodynamic limit, provides mechanistic insights into the learning processes generating desired neural representational geometries and generalization. This includes the role of weight norm regularization, network architecture, and hyper-parameters. We will explore recent advances in this theory and ongoing challenges. We also discuss the dynamics of learning and its relevance to the issue of representational drift in the brain.

Humans and animals exhibit a remarkable ability to generalize from limited experiences to novel situations. This trait is likely related to the ability of the neuronal system to extract from the stream of complex noisy high dimensional input signals features which are relevant for downstream computation, a property known as “feature learning”. Understanding the generalization and feature learning in biological neural networks can lead to significant breakthroughs in both neuroscience and artificial intelligence (AI) (1).

In recent years, AI has undergone a transformative advancement in capability, primarily fueled by developments in DNNs (2). These computational models have achieved unparalleled success across diverse domains, ranging from image recognition and natural language processing to structural biology and medicine. Broadly, their exceptional performance is rooted in their ability to generalize from training data to unseen inputs. Although the generalization power of DNNs falls short of human brains, understanding the mechanisms behind generalization in DNNs could provide insights into the principles governing generalization in neural networks within the brain. The study of learning in DNNs offers an important opportunity to understand the process of feature learning in complex learning systems.

In DNNs, data representation undergoes iterative refinement through multiple layers, capturing increasingly abstract features, yielding a top layer (“the feature layer”) whose representations serve as substrates for a broad spectrum of downstream computations (3).

This Perspective explores the impact of neural representations on the generalization capabilities of artificial and brain neural networks. One facet of generalization is to predict the correct response on a learned task for novel “test inputs” which are sampled from the same distribution as the training examples (in-distribution generalization). A more challenging capability is to rapidly learn new tasks. The example we will explore is that of few-shot learning, where the trained network is capable of learning new tasks using few examples (4). We will elucidate the role of the learned representations in each of these capabilities.

For decades, neuroscientists have studied single neurons’ receptive fields and tuning curves across various sensory arrays (e.g., in the retina, the cochlea, and the olfactory receptor neurons) as well as in sensory and motor cortices, e.g., primary visual area (V1), primary somatosensory area (S1), and primary motor area (M1). The difficulties in extending this program beyond primary areas suggest that understanding neural representations in higher stages of processing requires population-level theoretical and experimental approaches. An increasingly powerful line of research focuses on the topological and geometrical properties of an ensemble of population responses, known as neural manifolds (5–11). In the first part of this paper, we will demonstrate the successful applications of geometric approaches in predicting key aspects of generalization in the context of object recognition tasks. In the second part of the paper, we will explore our current understanding of the relation between generalization and the emergence of these representations in DNNs.

## Neural Representational Geometry underlying Object Recognition

### Geometry and Separability of Object Manifolds.

**Fig. 1.**
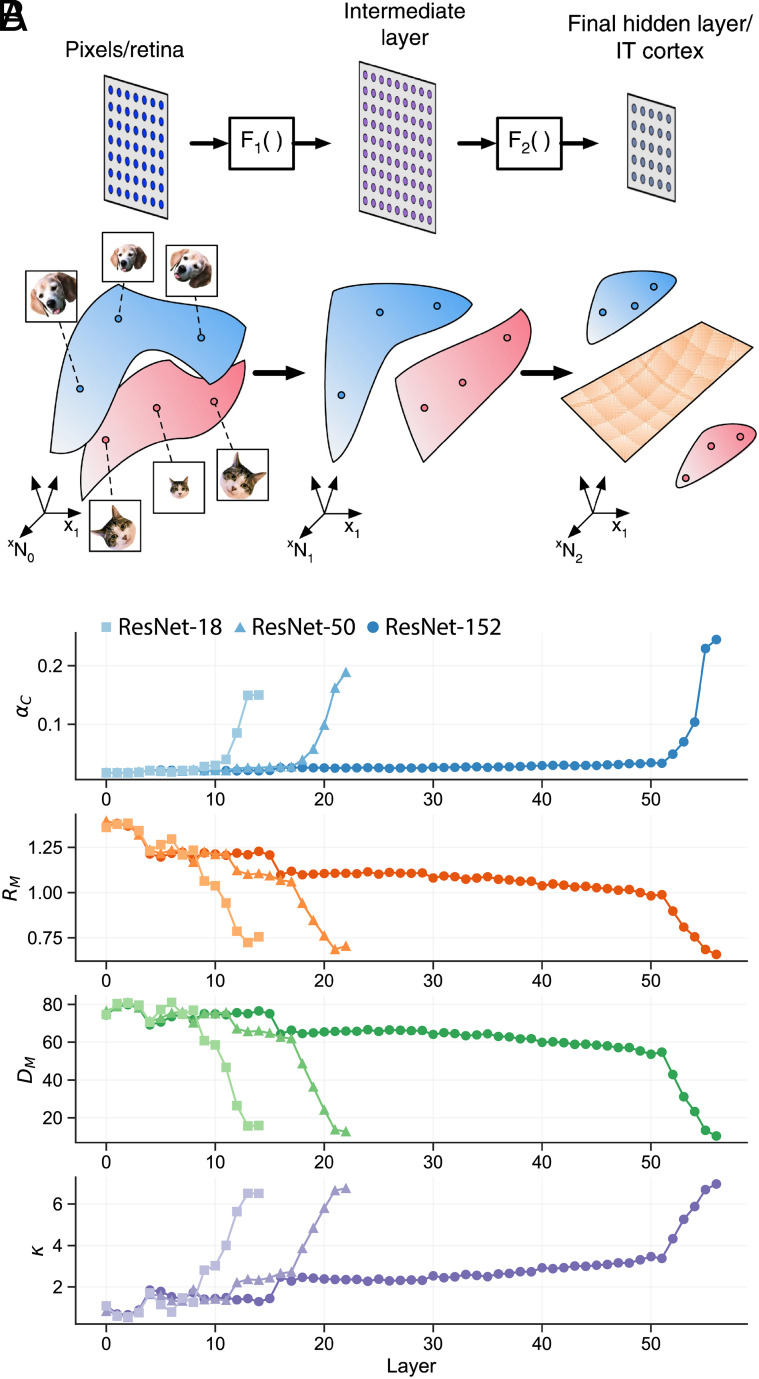
(*A*) Illustration of three layers in a visual hierarchy where the population response of the first layer is mapped into intermediate layer by F1 and into the last layer by F2 (*Top*) (10). The transformation of per-stimuli responses is associated with changes in the geometry of the object manifold, the collection of responses to stimuli of the same object (colored blue for a “dog” manifold and pink for a “cat” manifold). Changes in geometry may result in transforming object manifolds that are not linearly separable (in the first and intermediate layers) into separable ones in the last layer (separating hyperplane, colored orange). (*B*) Changes in classification capacity $\alpha_{C}$, manifold radius $R_{M}$, manifold dimension $D_{M}$, and classification margin κ across the layers of pre-trained DNNs (ResNets).

The set of neural population responses to stimuli belonging to the same object defines an *object manifold*. Intuitively, to perform well on object identity tasks, object manifolds at the top stages of the visual hierarchy (IT cortex in the ventral visual stream) should be well separated from each other (12). How do we quantify the degree to which object manifolds satisfy this property? A simple approach is to consider random binary classification tasks and check whether the tasks can be performed by a linear classifier downstream of a given neuronal layer. The utility of the object manifolds can be quantified by the maximum number of objects that can be classified with high probability by a separating hyperplane in the population state space (Fig. 1*A*).

The classical theory of linear classification, often limited to finite, weakly correlated input vectors, is inapplicable for the problem of classifying manifold data. To close this gap, we have developed a statistical mechanics theory of linear separability of manifolds (13). Our theory identifies three key metrics: manifold dimension $D_{M}$, radius $R_{M}$, and inter-manifold correlation ρ as the primary determinants of manifold separability. We consider a layer consisting of N neurons responding to numerous images belonging to P objects, forming P object manifolds; the system’s load is defined by the ratio α=P/N. We ask whether these manifolds can be separated into two randomly labeled classes by a hyperplane. In the regime where P and N are large, our theory shows the existence of a critical load value $\alpha_{c}$, called manifold classification capacity, such that when $P<\alpha_{c}N$ object manifolds are linearly separable with high probability, whereas if $P>\alpha_{c}N$ the manifolds are inseparable with high probability. Intuitively, this capacity serves as a measure of the amount of linearly decodable information per neuron about object identity. Thus, the theory predicts that the total number of objects which can be well-represented is extensive, proportional to the total number of neurons participating in the representation.

Next, we study how the shapes of the manifolds determine the value of $\alpha_{c}$. As stated above, theory predicts that the key metrics are their radius and dimensionality. The first measures the overall extent of the manifolds (relative to the distance between their centers) and the second measures the number of directions that these manifolds span.

As shown in ref. 13, the well-known concept of support vectors can be generalized to manifolds, where the weight vector normal to their separating plane is a linear combination of anchor points. Each manifold contributes (at most) a single anchor point, residing in the manifold or its convex hull. These points uniquely define the separating plane, thus anchoring it. The identity of the anchor points depends not only on the manifolds’ shape but also on their location or orientation in the N dimensional state space as well as the particular choice of random labeling. Thus, for a given fixed manifold, as the location and labeling of the other manifolds are varied, the manifold’s anchor point will change, thereby generating a distribution of the its anchor points. The manifold’s radius $R_{M}$ is the total variance of its anchor points normalized by the average distance between the manifold centers. Its dimension $D_{M}$ is the spread of the anchor points along the different manifold axes. The mean-field theory provides precise algorithms for estimating these quantities for any given set of manifolds (9, 13).

For manifolds spanning D≫1 dimensions, the classification capacity is well approximated by $R_{M}$ and $D_{M}$ through

[1] $$\begin{array}{c} \alpha_{C}=D_{M}^{-1}\left(1+R_{M}^{-2}\right). \end{array}$$

The theory predicts that when $R_{M}\sqrt{D_{M}}<1$ capacity is of O(1) whereas when $R_{M}\sqrt{D_{M}}\gg 1$ the manifolds are “entangled” yielding a capacity of O(1/D) (note that in the entangled regime $D_{M}\approx D\gg 1$). This theory assumes that the positions and orientations of different manifolds are uncorrelated. Object manifolds induced by real images show substantial correlations between their positions (i.e., their centers) particularly in early stages of the deep hierarchy. These correlations exhibit prominent low rank structure. Hence, the correlations can be accounted for by projecting all the points in the P manifolds (at each layer) to the null space of the center-center correlations. Recent work extends this theory to the case where not only the manifolds’ centroids, but also their directions of variability are correlated (14).

We have applied this framework to the study of the geometry of neural representations of object manifolds in DNNs pre-trained for object recognition tasks on large labeled dataset, ImageNet (15), including AlexNet (16), VGG (17), and ResNet (18). In each network, we measure classification capacity and geometry of point-cloud manifolds generated by responses to high-scoring samples from ImageNet classes (15) in each layer (10). Results of this analysis (shown in Fig. 1*B* for ResNet) demonstrate that the manifold classification capacity increases along the hierarchy of a fully trained deep network, with a concomitant decrease in manifold dimension and radius. Across most of the stages, the reduction in dimension and radius are incremental followed by steep changes in the last stages; a pattern that is apparent in other architectures as well (10).

While the classification capacity does not directly provide information about generalization, in particular, the likelihood that the system trained on a set of images would correctly classify held-out images from the same classes (“test accuracy”), we can use the notion of max-margin from the theory of support vector machines (SVM) (19) as a good proxy for the test accuracy. Similar to the margin in SVMs, the margin in our context is the distance of the “anchor points” from the separating plane that is optimized to maximize this distance. Naturally, if the load α is near capacity, the margin is close to zero. For a fixed load α below capacity, the maximum achievable margin is given in terms of the manifold radius and dimensionality, by

[2] $$\begin{array}{c} \kappa \left(\alpha \right)=\sqrt{\alpha^{-1}{\left(1+R_{M}\right)}^{2}}-R_{M}\sqrt{D_{M}}. \end{array}$$

The behavior of the manifold margin is shown in the *Bottom* plot of (Fig. 1*B*). Here the value of α is fixed to a value close to the minimal capacity, yielding almost zero margin at the early stages. Overall, there is a large 7-fold increase in margin value from the pixel layer to the feature layer.

### Manifold Geometry for Few-Shot Category Learning.

**Fig. 2.**
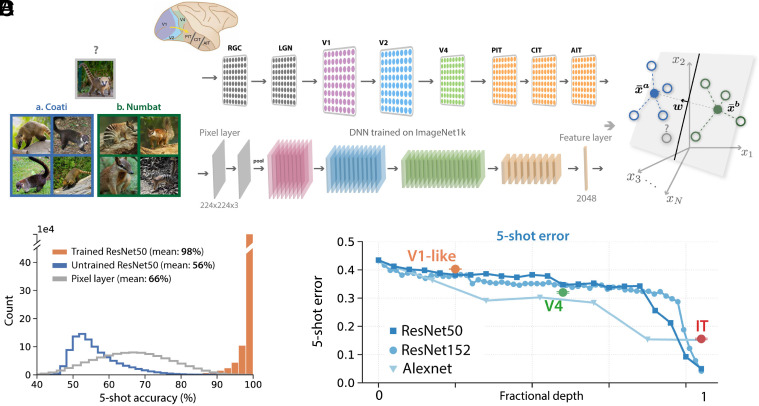
(*A* and *B*) Examples of novel objects, here “coatis” (blue) and “numbats” (green), are presented to the ventral visual pathway (*Top*), modeled by a trained DNN (*Bottom*), eliciting a pattern of activity across IT-like neurons in the feature layer. We model concept learning as learning a linear readout w to classify these activity patterns. (*C*), Generalization accuracy is very high across pairs of novel objects from the ImageNet21k dataset when using a pre-trained DNN (orange), but poor when using a randomly initialized DNN (blue), or a linear classifier in the pixel space of input images (gray). (*D*) Few-shot learning improves along the ventral visual hierarchy from pixels to V1 to V4 to IT, due to orchestrated transformations of object manifold geometry. The layerwise behavior of a trained ResNet50 (blue), Alexnet (light blue), and an untrained ResNet50 (gray) is included for comparison. We align V1, V4, and IT to the most similar ResNet layer under the BrainScore metric (20) (see ref. 11 for details).

Another facet of generalization is the ability to transfer knowledge acquired during training to rapidly learn novel tasks. In the context of object recognition, we discuss few-shot learning, the ability to learn new object categories from just a few novel examples, building on established representations from learning a large number of other object categories. As schematized in Fig. 2 *A* and *B*, one or a few examples of two novel objects (here coatis and numbats) are presented, and are mapped through the layers of the ventral visual stream of a mature animal (*Top*), or the layers of a DNN pre-trained for object recognition tasks (*Bottom*), resulting in high-dimensional neural representations of each example.

The few-shot learning happens only downstream, and is modeled by a single readout neuron learning a decision boundary between two novel objects on the basis of these few examples (Fig. 2*B*, *Right*). Several common choices of linear and nonlinear decision rules (e.g., SVMs, nearest neighbor classifiers) all match or underperform a simple linear classifier trained with a prototype-learning rule: averaging the few examples of each class into a central “prototype,” serving as an approximation of the true prototype of the new category manifold, and classifying a new input according to which of the two estimated “prototypes” is closer to it.

In experiments on pre-trained DNNs (details in *SI Appendix*, 1*A*), we find that with the simple prototype learning rule, these pre-trained representations are powerful enough to support good few-shot learning performance (Fig. 2*C*). Furthermore, performance consistently improves along the layers of the pre-trained DNN (Fig. 2*D*).

We additionally perform numerical experiments on neural representations in the visual cortices of primates (21) (details in *SI Appendix*, 1*B*). We find these representations are also powerful enough for few-shot learning performance of visual objects, and that few-shot performance improves along the visual hierarchy (Fig. 2*D*).

To understand what features of the neural representation empower good few-shot learning performance, we introduce a mathematical theory relating few shot learning of new objects to the geometry of their underlying manifolds. Unlike the complex manifold geometry governing object classification capacity, an ellipsoidal approximation of their geometry is sufficient to account for few shot prototype learning. Thus, performance is well predicted by each (true) manifold’s centroid $x_{0}$, and radii $R_{i}$ along a set of orthonormal basis directions $u_{i},i=1,\ldots ,N$, capturing the extent of natural variation of examples belonging to the same object. A useful measure of the overall size of these variations is the mean squared radius $R^{2}\equiv \frac{1}{N}\sum_{i=1}^{N}R_{i}^{2}$. The reason for this simplified geometry is that unlike the case of separating a large number of manifolds each consisting of a large (or infinite) number of points, here, the separating plane is determined only by the empirical centroids and does not have access to the more salient manifold statistics such as the anchor points.

Our theory predicts that the average error of m-shot learning on test examples of object a is given by $\varepsilon_{a}=H\left({\text{SNR}}_{a}\right)$, where H(·) is the Gaussian tail function $H\left(x\right)=\int_{x}^{\infty }dt$ $e^{-t^{2}/2}/\sqrt{2\pi }$. The quantity $\;{\text{SNR}}_{a}$ is the signal-to-noise ratio (SNR) for manifold a, whose dominant terms are given by,

[3] $$\begin{array}{c} \;{\text{SNR}}_{a}=\frac{1}{2}\frac{{\left\|\Delta x_{0}\right\|}^{2}+\left(R_{b}^{2}R_{a}^{-2}-1\right)/m}{\sqrt{D_{a}^{-1}/m+\|\Delta x_{0}\cdot U_{b}{\|}^{2}/m+{\left\|\Delta x_{0}\cdot U_{a}\right\|}^{2}}}. \end{array}$$

A full expression and derivation is given in ref. 11. The SNR depends on four interpretable geometric properties:

(1) Signal. ${\left\|\Delta x_{0}\right\|}^{2}\equiv {\left\|x_{0}^{a}-x_{0}^{b}\right\|}^{2}/R_{a}^{2}$ represents the pairwise distance between the manifolds’ centroids, ${x_{0}}^{a}$ and ${x_{0}}^{b}$, normalized by $R_{a}^{2}$. Well-separated manifolds have a higher SNR, and hence a lower generalization error.
(2) Bias. $R_{b}^{2}R_{a}^{-2}-1$ represents the average bias of the linear classifier. Importantly, this bias is asymmetric: When manifold a is larger than manifold b, the bias term is negative, predicting a lower SNR for manifold a.
(3) Dimension. A natural notion of dimensionality arises in our theory, known as the participation ratio $D_{a}\equiv {\left(R_{a}^{2}\right)}^{2}/\sum_{i=1}^{N}{\left(R_{i}^{a}\right)}^{4}$, which quantifies the number of dimensions along which the object manifold varies significantly, and is often much smaller than the number of neurons *N*. This is the analog of manifold dimension $D_{M}$ studied above. However, in contrast to the role of dimensionality for capacity discussed above, Eq. **3** reveals that for few-shot learning, high-dimensional manifolds are preferred.
(4) Signal-noise overlap. $\|\Delta x_{0}\cdot U_{a}{\|}^{2}$ and $\|\Delta x_{0}\cdot U_{b}{\|}^{2}$ quantify the overlap between the signal direction $\Delta x_{0}$ and the manifold axes of variation $U_{a}\equiv \left[u_{1}^{a}R_{1}^{a},\ldots ,u_{N}^{a}R_{N}^{a}\right]/\sqrt{R_{a}^{2}}$ and $U_{b}\equiv \left[u_{1}^{b}R_{1}^{b},\ldots ,u_{N}^{b}R_{N}^{b}\right]/\sqrt{R_{b}^{2}}$. Generalization error increases as the overlap between the signal and noise of directions increases. We note that signal-noise overlap is bounded above by $1/D_{a}$, and hence is small in high dimensions.

**Fig. 3.**
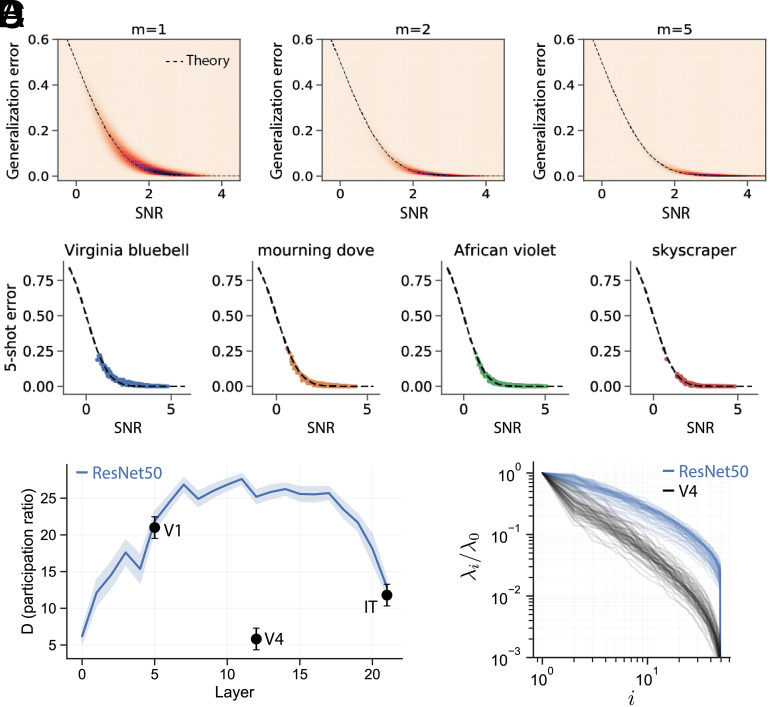
(*A*) We compare the empirical generalization error in 1-, 2-, and 5-shot learning experiments to the prediction from our geometric theory (Eq. **3**) on all pairs of objects from the ImageNet21k dataset, using object manifolds derived from a trained ResNet50. x-axis: SNR obtained by estimating neural manifold geometry. y-axis: Empirical generalization error measured in few-shot learning experiments. Theoretical prediction (dashed line) shows a good match with experiments. (*B*) We provide additional examples of 5-shot prototype learning experiments in a ResNet50 (colored points), along with the prediction from our geometric theory (dashed line), on four randomly selected novel visual objects from the ImageNet21k dataset. Each panel plots the generalization error of one novel visual object (e.g., “Virginia bluebell”) against all 999 other novel visual objects. Each point represents the average generalization error on one such pair of objects. x-axis: SNR (Eq. **3**) obtained by estimating neural manifold geometry. y-axis: Empirical generalization error measured in few-shot learning experiments. Theoretical prediction (dashed line) shows a good match with experiments. (*C*) In a pre-trained ResNet50 (blue) dimensionality expands dramatically in the early layers and contracts in the later layers, while in the primate visual pathway (Black) dimensionality contracts from the V1-like layer to V4, then expands from V4 to IT. (*D*) Single-manifold eigenspectra in macaque V4 (black) and the corresponding layer of a pre-trained ResNet50 (blue).

To validate our theory, we conducted experiments on visual object manifolds from pre-trained DNNs (ResNet50) and primate IT cortex neural activity (21), finding agreement across visual categories (Fig. 3 *A* and *B*).

### Comparing Geometry in DNNs and the Primate Visual Pathway.

While the SNR increases along both the primate visual hierarchy and the successive layers of pre-trained DNNs (Fig. 2*D*), the individual underlying geometric quantities may show different behavior. In particular, the dimension of object manifolds expands dramatically in the early layers of trained DNNs, and compresses in the final layers (Fig. 3*C*). This dimensionality expansion and compression has been observed in other recent works and architectures (23, 24). In contrast, the dimension of object manifolds in the primate visual pathway remains low throughout V4 and IT cortex (Fig. 3*C*). This difference is highlighted in Fig. 3*D*, which shows that the eigenspectra of object manifolds in V4 are low dimensional, and well described by a power law, while the eigenspectra in the corresponding layer of a trained DNN are much higher dimensional. Future work could explore the computational underpinnings of these differences.

### Comparing Geometry of Vision and Language Representations.

Our finding that downstream classifier can use empirical prototypes obtained by few-shot learning raises the question whether information from other modalities may also be used to approximate vision prototypes in the feature layer, enabling transfer learning of new categories. Indeed in ref. 11, we find a surprising alignment between representations in vision models pre-trained on images and word vector embedding models pre-trained on text. Object prototypes in the visual embedding space and their corresponding language representations can be closely aligned by a rotation operation. Moreover, we show that this alignment generalizes to novel objects, so that new visual categories can be correctly discriminated purely by a language-based descriptor (“zero-shot” learning). This finding suggests that the two pre-training processes endow vision and language models with a similar fine-grained, generalizable semantic structure. This conclusion is supported by the finding that the geometry of visual representations encodes a rich hierarchical structure (11), *SI Appendix*, Fig. 1. Interesting recent works have investigated the structure and origin of this hierarchical structure (25, 26).

## Theory of Deep Learning

We have discussed the geometric properties of neural manifolds that are necessary and sufficient for good generalization capabilities. To understand the learning mechanisms that give rise to these representations requires a theory of how learning in deep networks shapes neural representations. In this section, we review recent advances in the theory of fully connected deep wide networks (27–39). We will compare the predictions of these theories regarding the geometry of learned representations against our results on object manifolds, suggesting future directions of analyzing feature learning in more complex DNNs.

### Sampling the Space of Solutions.

Wide DNNs are examples of over-parameterized neural networks, in which the training data can be perfectly fit by many choices of weights, only a subset of which yields good generalization. One strategy to sample solutions with good generalization performance is to bias the sampling to solutions with small weight norms, as they tend to mitigate overfitting (40, 41). Recent DNN theories focus on two disparate implementation schemes. One approach focuses on learning by gradient descent (GD) on the training cost function, where different solutions are reached by varying the initialization. In this case, weight norms are controlled indirectly by the norms of the initialized ones. Performance of GD also depends on details of the learning dynamics, including batch sizes, learning rates, and initialization (36, 42–44). The second approach focuses on Bayesian neural networks (BNNs) (45) where the effect of learning is characterized by a posterior distribution in weight space. Sampling of weights from this posterior distribution determines the statistics of the input–output function of the network. Significant analytical progress has been made in both approaches in the limit where the width of the network is large.

### Predictor, Loss Function and Generalization Error.

In a fully connected DNN, for a given set of weights and an input $x\in R^{N_{0}}$, the output, called the predictor, is given by a linear summation of the activation of the last hidden layer (the “feature layer”):

[4] $$\begin{array}{c} f\left(x,\Theta \right)=N^{-\gamma }\Phi {\left(W,x\right)}^{⊤}a\in R^{1\times M}, \end{array}$$

where M is the dimension of the output, and a is the linear readout weight. Different choices of γ may result in vastly different behaviors as discussed in later sections. One choice, common in practice and in theoretical investigations, known as the “lazy regime”, is γ=1/2. This is the regime we’ll focus on in this perspective, as opposed to the “nonlazy” regime, where γ=1 (46). We denote the network parameters as Θ={W,a}, with the hidden layer weights W and readout weights a. Φ(W,x) is the vector of responses of the “feature layer” to an input vector x, $\Phi \left(W,x\right)=\phi (h^{L}\left(x\right))$ where the pre-activations of the l-th layer, l=2,⋯,L, are defined as $h^{l}\left(x\right)=N^{-\frac{1}{2}}W^{l}\cdot \phi \left(h^{l-1}\left(x\right)\right)$, and $h^{1}\left(x\right)=N_{0}^{-\frac{1}{2}}W^{1}\cdot x$. Here $W={\left\{W^{l}\right\}}_{l=1}^{L}$ and $W^{2},\cdots ,W^{L}\in R^{N\times N}$, $W^{1}\in R^{N\times N_{0}}$, where N denotes the width of all hidden layers. The function ϕ(·) denotes the nonlinear activation function. For simplicity, we use the squared error (SE) loss both during training and for evaluating test performance. Denoting the set of P training data points as $D={\left\{X_{\mu },Y_{\mu }\right\}}_{\mu =1,\cdots ,P}$, $X\in R^{P\times N_{0}},Y\in R^{P\times M}$, DNNs trained with GD minimizes

[5] $$\begin{array}{c} L\left(D,\Theta \right)=\frac{1}{2}\sum_{\mu =1}^{P}||f\left(X_{\mu },\Theta \right)-Y_{\mu }{\left|\right|}^{2}, \end{array}$$

starting from some random initialization $\Theta_{0}$ and performing weight updates in proportion to $-\nabla_{\Theta_{t}}L\left(D,\Theta_{t}\right)$. Initial weights are chosen from an iid Gaussian distribution $\Theta_{0}∼N\left(0,\sigma_{0}^{2}I\right)$. In contrast, BNNs sample from the posterior distribution

[6] $$\begin{array}{c} P\left(\Theta |D\right)\propto P_{0}\left(\Theta \right)P\left(D|\Theta \right)\propto P_{0}\left(\Theta \right)\exp\left(-\beta L\left(D,\Theta \right)\right), \end{array}$$

where we choose a Gaussian prior $P_{0}\left(\Theta \right)\propto \;\exp({\left(2\sigma^{2}\right)}^{-1}{\left||\Theta |\right|}^{2})$. β is the inverse temperature $\beta =T^{-1}$ controlling the relative strength of the likelihood P(D|Θ) over the prior. Hereafter we focus on the limit β→∞ which constrains the posterior distribution within the L(D,Θ)=0 solution space. The generalization error per input x with ground truth label y(x) can be decomposed into bias and variance components

[7] $$\begin{array}{c} \epsilon_{g}\left(x,y\left(x\right)\right)=\overset{\mathrm{bias}}{\overbrace{{||\left\langle f\left(x,\Theta \right)\right\rangle }_{\Theta }-y\left(x\right){\left|\right|}^{2}}}+\overset{\mathrm{variance}}{\overbrace{{\left\langle ||\delta f\left(x,\Theta \right)|\right|}^{2}\rangle_{\Theta }}}, \end{array}$$

where ${\left\langle \cdot \right\rangle }_{\Theta }$ denotes averaging over the posterior distribution Eq. **6**. Thus, the mean and variance of the predictor determine the generalization error.

### Predictor Statistics and Kernel Functions.

Using the BNN model, Eq. **6**, it is straightforward to average over the readout weights a conditioned on W, yielding

[8] $$\begin{array}{c} {\left\langle f\left(\Theta ,x\right)\right\rangle }_{a}=k_{L}{\left(x\right)}^{⊤}K_{L}^{-1}Y, \end{array}$$

[9] $$\begin{array}{c} {\left\langle \delta f{\left(x,\Theta \right)}^{⊤}\delta f\left(x^{\prime },\Theta \right)\right\rangle }_{a}=I_{M}\cdot \left(K_{L}\left(x,x^{\prime }\right)-k_{L}{\left(x\right)}^{⊤}K_{L}^{-1}k_{L}\left(x^{\prime }\right)\right), \end{array}$$

where ${\left\langle \cdot \right\rangle }_{a}$ denotes partial averaging over the conditional distribution P(a|W,D), and $I_{M}$ denotes an M×M identity matrix. These statistics are given in terms of the top layer kernel function $K_{L}$ (Eq. **10**). For each layer, $K_{l}$ is a scalar function of a pair of inputs $\left\{x,x^{\prime }\right\}$ (27)

[10] $$\begin{array}{c} K_{l}\left(x,x^{\prime }\right)\equiv \frac{\sigma^{2}}{N}\phi \left(h^{l}\left(x\right)\right)\cdot \phi \left(h^{l}\left(x^{\prime }\right)\right),l=1,\cdots ,L. \end{array}$$

From this function, the P×P data kernel matrix is constructed as $K_{l}^{\mu \nu }=K_{l}\left(X^{\mu },X^{\nu }\right)$ and the P×1 kernel vector is defined as $k_{l}^{\mu }\left(x\right)=K_{l}\left(x,X^{\mu }\right)$. These kernel functions depend on the hidden layer weights W; averaging over them is highly nontrivial due to the non-Gaussianity of W. How to make tractable this average in different regimes remains a challenging question, as we discuss in detail below.

### Infinitely Wide DNNs.

Infinitely wide networks have been the target of numerous theoretical studies (27–33). The infinite width limit is defined as taking the dimensionality of the hidden layers, N, to infinity while keeping the size of the training dataset, P, finite. For instance, in the BNN model of Eq. **6**, the first- and second-order predictor statistics in this limit are given by

[11] $$\begin{array}{c} {\left\langle f\left(x,\Theta \right)\right\rangle }_{\Theta }=k_{L}^{\mathrm{GP}}{\left(x\right)}^{⊤}{K_{L}^{\mathrm{GP}}}^{-1}Y, \end{array}$$

[12] $$\begin{array}{c} {\left\langle \delta f{\left(x,\Theta \right)}^{⊤}\delta f\left(x^{\prime },\Theta \right)\right\rangle }_{\Theta }=I_{M}\cdot \left(K_{L}^{\mathrm{GP}}\left(x,x\right)-k_{L}^{\mathrm{GP}}{\left(x\right)}^{⊤}{K_{L}^{\mathrm{GP}}}^{-1}k_{L}^{\mathrm{GP}}\left(x\right)\right). \end{array}$$

Here, the W-dependent kernel functions of Eqs. **8**–**10** are replaced by their averages over the Gaussian prior of W,

[13] $$\begin{array}{c} K_{l}^{\mathrm{GP}}\left(x,x^{\prime }\right)\equiv {\left\langle K_{l}\left(x,x^{\prime }\right)\right\rangle }_{W∼N(0,\sigma^{2}I)} \end{array}$$

and thus no longer depend on W. This kernel function is referred to as the Neural Network Gaussian Process (NNGP) kernel. Similarly, the corresponding P×P data kernel matrix and P×1 kernel vector are constructed by $K_{l}^{GP,\mu ,\nu }=K_{l}^{\mathrm{GP}}\left(X^{\mu },X^{\nu }\right)$ and $k_{l}^{GP,\mu }\left(x\right)=K_{l}^{\mathrm{GP}}\left(x,X^{\mu }\right)$, respectively. The NNGP kernels can be calculated iteratively across layers, and for some choices of the nonlinearity ϕ(·), analytical forms of this recursion relation have been derived (27).

Expressions similar to Eqs. **11** and **12** hold for GD-trained infinitely wide DNNs but with a different kernel function, the neural tangent kernel (NTK) (28, 33). Quantitative differences between the NTK and the NNGP have been systematically studied for different network architectures and tasks (47). However, the connection between the two frameworks has not yet been elucidated. In the following section, we describe our recent work (39) which unifies them.

### Langevin Learning Connects GD Training and BNNs.

Unlike learning by GD, the BNN formulation does not specify the learning dynamics for sampling from the posterior Eq. **6** and various efficient sampling methods have been proposed (48, 49). Here, we consider sampling by Langevin dynamics, a gradient-based stochastic dynamical process which at long times corresponds to sampling from Gibbs equilibrium distribution (50). In our case, the dynamics of the network parameters Θ take the form

**Fig. 4.**
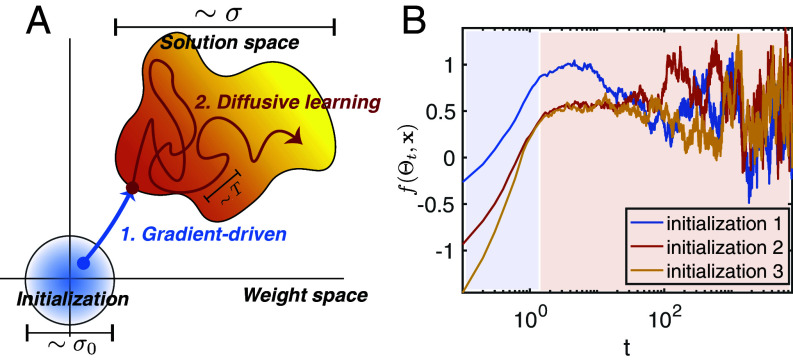
(*A*) Two stages of learning of Langevin dynamics with small *T*, $\sigma_{0}$ controls the width of weight distribution at initialization, σ controls the size of the solution space, and T relates to the sampling speed. (*B*) Example trajectories of the predictor from three different initializations, the dynamics is initially deterministic and starts to fluctuate as $\Theta_{t}$ drifts in the solution space after reaching zero training error.

[14] $$\begin{array}{c} \frac{d\Theta_{t}}{\mathrm{dt}}=-\nabla_{\Theta }L\left(\Theta_{t},D\right)-T\sigma^{-2}\Theta_{t}+\eta_{t}, \end{array}$$

where $\eta_{t}$ is Gaussian white noise with zero mean and covariance $\left\langle \eta_{t}\eta_{t}^{\prime ⊤}\right\rangle =2T\delta \left(t-t^{\prime }\right)I$. The weight decay term is the gradient of an $L_{2}$ weight norm regularization, or equivalently the exponent of the prior $P_{0}\left(\Theta \right)$. When T=0, the above dynamics is deterministic and corresponds to continuous time GD. For any finite *T*, the process converges to sampling from the posterior distribution, Eq. **6**. Here we describe the interesting regime where T is small but nonzero (51). By analyzing the distribution of the dynamical trajectories induced by the above Langevin dynamics (Eq. **14**) at small *T* in the infinite width limit, we are able to characterize the dynamics that connects the deterministic GD learning (short times) to sampling from the BNN posterior (long times), recovering both NTK and NNGP results under different time scales. Fig. 4 *A* and *B* offer an intuitive illustration of the dynamic process. The dynamics initially approximate the GD dynamics, as the first term on the RHS of Eq. **14** dominates. We refer to this learning stage as gradient-driven phase. As the training error reaches approximately 0, the gradient contribution becomes the same order as the noise $\eta_{t}$, entering what we refer to as the diffusive phase. The predictor fluctuates significantly as the dynamics explore the solution space driven by small noise. Although the initial gradient-driven stage largely depends on initialization as in GD, the dynamics become ergodic in the diffusive phase. When t scales as $\sigma^{2}/T$ (σ controls size of the solution space and T controls the speed of exploration), the predictor statistics averaged across time becomes independent of initialization, as expected for BNNs.

Our formulation allows for investigation of how representations and generalization performance vary during the initial transient and the gradual exploration of the solution space.

### Finite Width Kernel Renormalization.

Comparing the infinite width limit predictions to real wide neural networks with $N∼10^{2}$ to $10^{3}$ requires restriction to relatively small number of examples P, often failing to capture the properties of realistic networks where both N and P are large. A more realistic regime is one where the number of training examples scales linearly with the network width, namely $P\to \infty ,N,N_{0}\to \infty ,\alpha \equiv \frac{P}{N},\alpha_{0}\equiv \frac{P}{N_{0}}∼O\left(1\right)$. We refer to this regime as the thermodynamic limit. In this section, we discuss our results in this regime (38), focusing on the BNN posterior framework.

**Fig. 5.**
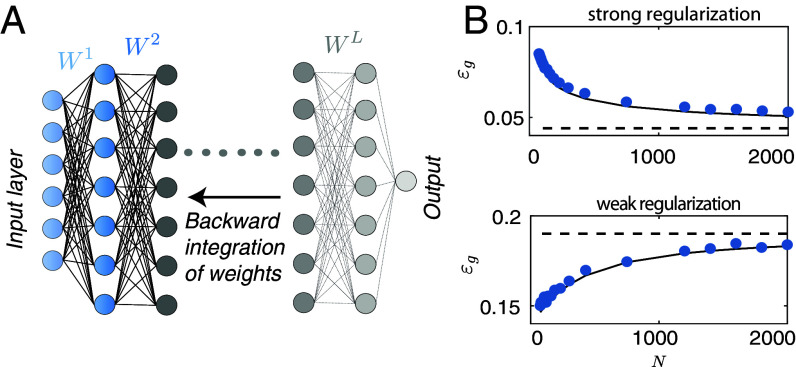
(*A*) Schematics for the BPKR approach. A renormalization factor is introduced at each step during backward integration until all the network weights are averaged out. (*B*) Theory (black solid line) and simulation (blue points) of generalization error $\varepsilon_{g}={\left\langle \epsilon_{g}\left(x,y\left(x\right)\right)\right\rangle }_{\left\{x,y(x)\right\}}$ on binary MNIST classification in fully connected ReLU networks, for small (*Top*) and large (*Bottom*) σ. The approximate theory for ReLU networks agrees remarkably well with the numerics.

Although the prior on the weights is Gaussian, the constraints imposed by the likelihood term on the training data cannot be ignored in the finite α regime. We show that in this regime the effect of finite α can be expressed in terms of a kernel renormalization, which can be derived using the Back-Propagating Kernel Renormalization (BPKR) procedure. The BPKR approach allows us to integrate the network weights in a backward direction, starting from the readout weights a, and proceeding to $W^{L},W^{L-1},\cdots ,W^{1}$ , as shown in Fig. 5*A*. At each stage of the integration, we introduce a renormalization factor, which summarizes the effect of the integrated weights. After averaging over all weights, we find that the predictor statistics still follow the same form as Eqs. **11** and **12**, but with the NNGP kernel function (Eq. **13**) replaced by a renormalized kernel function. For a network with single readout M=1 and L hidden layers, the renormalized kernel function is given by $\tilde{K}\left(x,x^{\prime }\right)={\left(\sigma^{-2}u_{0}\right)}^{L}K_{L,GP}\left(x,x^{\prime }\right)$. The renormalization factor $u_{0}$ is determined self-consistently by $u_{0}=\left(1-\alpha \right)\sigma^{2}+\sigma^{2}Y^{⊤}{\tilde{K}}^{-1}Y/P$. Here we use $\tilde{K}\in R^{P\times P}$ to denote the data kernel matrix constructed by ${\tilde{K}}^{\mu ,\nu }=\tilde{K}\left(X^{\mu },X^{\nu }\right)$. Note that at α=0, $u_{0}=\sigma^{2}$, reducing to the NNGP theory. Unlike the NNGP kernel function, this renormalized kernel function also depends on the target labels Y, reflecting the effect of training data on the posterior. Furthermore, $u_{0}$ can be related to the average norm of the readout weights a w.r.t. the posterior distribution, $u_{0}=\langle N^{-1}{\left||a|\right|}^{2}\rangle_{\Theta }$. Intuitively, the renormalization by $u_{0}$ captures how the learned partial alignment between the readout weights and the target labels affects the average readout weight norm, and in turn the predictor statistics.

For networks with multiple outputs, the renormalized kernel is given by the Kronecker product of the GP kernel with an M×M dimensional *renormalization matrix*$U_{0}$ (*SI Appendix*, C3 of ref. 38).

The mean predictor is unaffected by kernel renormalization because the renormalization cancels in the kernel and the inverse kernel. However, the predictor variance is affected. Importantly, we find that for strong norm regularization (small σ) the variance decreases with N, thus the infinite-width limit performance is optimal. Conversely, for large σ, error increases with the width, implying that the weak regularization fails to prevent overfitting as the network width increases (Fig. 5*B*). The transition point between the two regimes depends on the depth as well as the training data.

The above result is exact for linear networks in the thermodynamic limit. An interesting recent work (52) derived non-asymptotic expressions for the posterior predictor statistics and training data likelihood in terms of Meijer-G functions, and agrees with our kernel renormalization results in the thermodynamic limit. For ReLU networks the above kernel renormalization expressions are heuristic extension of the linear case. Surprisingly, we find that this approximation agrees remarkably well with the numerical simulations for ReLU networks as illustrated in Fig. 5*B*. The validity of this approximation is further discussed in recent works (53–56).

### Feature Learning in Wide Networks.

#### Mean layer-wise kernels.

Our BPKR framework allows for the computation of the changes in the representations in the network, which can be evaluated by the posterior average of the layer-wise kernel functions (Eq. **10**). We find that even in the thermodynamic limit, the mean kernels depart from their infinite width limit only by a correction of the order of 1/N. For instance, the P×P mean training data kernel matrix is,

[15] $$\begin{array}{c} {\left\langle K_{l}\right\rangle }_{\Theta }∼K_{l}^{\mathrm{GP}}+N^{-1}YG_{l}\left(U_{0}\right)Y^{⊤}, \end{array}$$

where $G_{l}\left(U_{0}\right)\in R^{M\times M}$ is a function of the renormalization matrix $U_{0}$. The first term is the NNGP kernel matrix which is generally full rank, and the second term is a rank M correction that aligns with the subspace spanned by the M×P-dimensional target labels Y. The expression for mean layer-wise kernel function on arbitrary test points are given in *SI Appendix*, 2*A*. We emphasize that the average kernel matrix ${\left\langle K_{l}\right\rangle }_{\Theta }$ is not equivalent to the renormalized kernel $\tilde{K}\left(x,x^{\prime }\right)$. The latter one appears in the predictor statistics which involves products and inverses of the hidden layer kernels as well as the effect of the posterior readout weights.

**Fig. 6.**
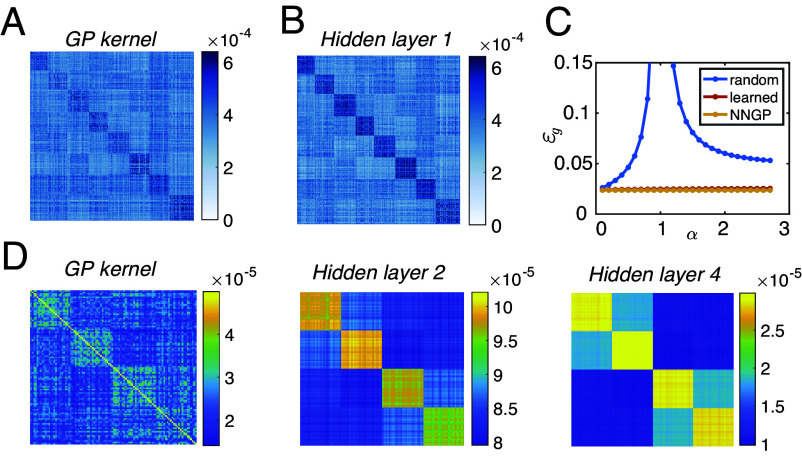
(*A* and *B*) NNGP and mean layer-wise kernels in classifying eight MNIST digits *SI Appendix*, 3, (57). (*C*) Generalization error averaged across test examples for finite width random feature model (blue), infinitely wide network following the NNGP theory (yellow), and the learned network following the BPKR theory (red, overlaying NNGP theory). (*D*) The NNGP kernel, and the mean layer-wise kernel of hidden layer l=2,4. For a 4-hidden-layer ReLU network trained on four MNIST digits grouped into two higher-order categories of even vs. odd. The values of the kernel are small since we take relatively small σ (*SI Appendix*, 3).

We note that the NNGP kernel scales as $\sigma^{2(l+1)}$, while $G_{l}\left(U_{0}\right)$ in the second term scales as $\sigma^{2}$. For σ<1, the first term shrinks rapidly as l increases while the magnitude of the second term remains unchanged, revealing a more pronounced learning-induced structure. Furthermore, in Eq. **15** the term $G_{l}\left(U_{0}\right)$ modifies the structure of the second term. In Fig. 6*D*, we show an example of our theory applied to an L=4 ReLU network, trained simultaneously on 4-way classification of four MNIST digits as well as on 2-way classification of even vs. odd. We see that learning-induced changes in the mean layer-wise kernel become more pronounced as we increase l. Furthermore, the structure of the kernel matrix changes across l due to the modification of $G_{l}\left(U_{0}\right)$; in particular, the higher order structure (the two larger blocks corresponding to even vs. odd) becomes more pronounced at the deeper layers.

#### Representation and generalization.

Although the learning-induced change in the mean layer-wise kernel is small, it is low rank and aligns with the network target output, therefore it may significantly affect the generalization performance. We investigate this effect by comparing the generalization error of a fully trained DNN with the predictor statistics given by the BPKR theory, to a DNN with random features Φ(W,x) ($W∼N(0,\sigma^{2}I)$) of the *same width*.

In Fig. 6*A*–*C* we show an example of a ReLU network with one hidden layer trained on MNIST classification (see details in *SI Appendix*, 3). As expected, the learned mean hidden layer kernel (Fig. 6*B*) exhibits slightly stronger block structure compared to the NNGP kernel (Fig. 6*A*). However, we see drastic improvement in the generalization performance in the trained DNNs compared to DNNs with random features across all values of α>0 (as shown in Fig. 6*C* red vs. blue lines). In particular, the generalization error diverges in the random feature model at α=1. This is because conditioned on any fixed random W, the norm of a diverges at α=1. This divergence does not show up in fully trained DNNs in which the hidden representations are partially aligned with the target outputs. Therefore, although feature learning in the thermodynamic limit is weak it allows the network to outperform the corresponding random feature model with the same finite hidden layer width, and yields a performance similar to the corresponding infinitely wide network captured by the NNGP theory (Fig. 6*C* red vs. yellow lines). This similar performance is because σ is chosen to be relatively small (σ=0.2), and the bias contribution dominates the generalization error.

### From Mean Kernels to Representational Geometry.

**Fig. 7.**
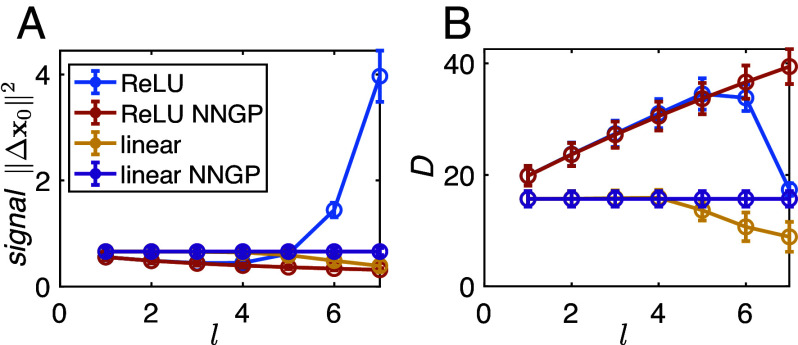
(*A*) Signal as a function of hidden layer depth l. For ReLU networks in the thermodynamic limit, signal increases with layer depth (blue). For linear networks (yellow, purple) and ReLU networks in the infinite width limit (red), signal remains unchanged across l. Error bars are across all distinct pairs of manifolds/digits. (*B*) Dimension as a function of l. In the infinite width limit, dimension remains constant with l in linear networks (purple) and increases with l in ReLU networks (red). In the thermodynamic limit, dimension decreases in linear networks (yellow), and is non-monotonic in ReLU networks (blue), similar to Fig. 3*C*. Error bars are across all manifolds/digits.

In the first part of this paper, we introduced several normative conditions on the representational geometry for obtaining good generalization performance in concept-identity tasks. Some of these measures can be readily obtained from the mean layer-wise kernels (*SI Appendix*, 2*B*). In Fig. 7, we present preliminary results for the signal $\left|\right|{\Delta x}_{0}{\left|\right|}^{2}$ and dimension $D_{a}$ defined in Eq. **3**. They are calculated from the mean layer-wise kernel functions (Eq. **15** and *SI Appendix*, 2*A*) and the NNGP kernel functions (Eq. **13**) for linear and ReLU networks (see details in *SI Appendix*, 2*B* and 3), applied on test samples from the MNIST dataset. Each manifold is determined by sample points from a unique digit. We compare these geometric measures across different hidden layers l. The signal increases monotonically with the layer depth, and the dimension is non-monotonic. These preliminary results, strikingly similar to the trends in the manifold geometry exhibited in DNNs trained for object recognition (Fig. 3*C*), suggest that the BPKR theory may provide a powerful theoretical tool to explain the emergence of category manifolds in deep networks. Extending these theoretical calculations to CNNs and to representations of held-out categories is a future direction.

### Representational Drift.

**Fig. 8.**
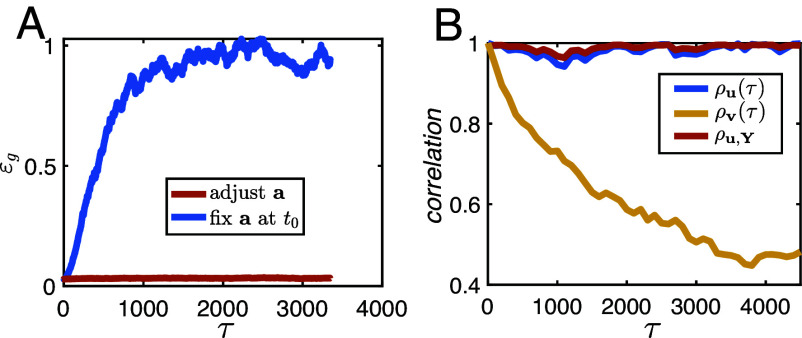
(*A*) Comparison of the generalization error dynamics between a network fully trained under Langevin dynamics (Eq. **14**, shown in red), and a network with a frozen at time $t_{0}$ in the diffusive learning stage, and $W_{t}$ randomly drifting afterward (shown in blue). τ denotes the difference between the current time t and $t_{0}$. (*B*) Both the temporal correlation of the top right singular vector ($\rho_{u}\left(\tau \right)$) and the correlation between u(t) and Y ($\rho_{u,Y}$) remain close to 1, representing the constant alignment between the *Top Right* singular vector of the representation and the training labels. Temporal correlation of the *Top Left* singular vector ($\rho_{v}\left(\tau \right)$) gradually decreases with the time difference τ, representing the random drift in the feature space.

Representational drift (RD) refers to neuroscience observations of neural activities accumulating changes over time without noticeably affecting the relevant animal behavior (58–62). It has been suggested that behavioral robustness to RD is due to readout changes that compensate for the drift in representational layers, maintaining a stable input–output relations (63, 64). Indeed, within our framework of learning under Langevin dynamics with small noise, the stability of the performance during the diffusion phase is due to the continuous realignment of readout weights $a_{t}$ to changes in $W_{t}$. Additionally, as shown above, the diffusion in $W_{t}$ is constrained by learning. Adhering to these constraints requires an ongoing learning signal. To highlight the importance of this signal, we consider an alternative scenario where the readout weights are frozen at some time (denoted as $t_{0}$) after achieving low training error while the weights of the hidden layers $W_{t}$ drift randomly without an external learning signal. As we show in Fig. 8*A*, while the generalization error remains small for Langevin learning dynamics (red), the performance degrades significantly in the absence of the learning signal (blue).

We seek to understand how the results on the mean layer-wise kernel translate to constrained drift of the representation $\phi (h^{l})$. Inspecting Eq. **15** we hypothesize that the dynamical trajectory of $\phi (h_{t}^{l}\left(X\right))$ can be approximately captured by

[16] $$\begin{array}{c} \phi \left(h_{t}^{l}\left(X\right)\right)=\phi \left(h^{l}\left(W_{t}^{0},X\right)\right)+N^{-1/2}z\left(t\right)Y^{⊤}, \end{array}$$

where $z\left(t\right)\in R^{N\times M}$ is a time-dependent random vector with mean 0 and $\left\langle N^{-1}z{\left(t\right)}^{⊤}z\left(t\right)\right\rangle =G_{l}\left(U_{0}\right)$. $W_{t}^{0}$ denotes a sample of the hidden layer weights from $N(0,\sigma^{2}I)$ and is independent of z(t), and $\phi (h^{l}\left(W_{t}^{0},X\right))$ denotes the l-th layer hidden activation on X with $W_{t}^{0}$. The hypothesis is consistent with Eq. **15**. The first term contributes to the NNGP kernel. The second term represents the representational drift within a space constrained by the task.

We have tested this hypothesis by simulating a single hidden layer, single output ReLU network, trained with Langevin dynamics (Eq. **14**). We track the hidden layer representations on the training data X during training. At time t, we denote the hidden layer activation data matrix as $\phi \left(h_{t}^{l}\left(X\right)\right)\in R^{N\times P}$. In order to characterize the drift, we compute the unit norm top right and left singular vectors of $\phi (h_{t}^{l}\left(X\right))$, denoted by $u\left(t\right)\in R^{P}$ and $v\left(t\right)\in R^{N}$ respectively, and track their temporal correlations in the diffusive learning stage. These temporal correlations are defined as $\rho_{u}\left(\tau \right)\equiv \;\lim_{t\to \infty }\left\langle u{\left(t+\tau \right)}^{⊤}u\left(t\right)\right\rangle$, and $\rho_{v}\left(\tau \right)\equiv \;\lim_{t\to \infty }\left\langle v{\left(t+\tau \right)}^{⊤}v\left(t\right)\right\rangle$. Furthermore, to quantify how the representation is constrained by the training labels Y, we define the correlation between u(t) and Y at equilibrium as $\rho_{u,Y}\equiv \;\lim_{t\to \infty }\left\langle u{\left(t\right)}^{⊤}Y\right\rangle /\left||Y|\right|$. As shown in Fig. 8*B*, we find that u(t) is constantly aligned with Y. Meanwhile, v(t) gradually decorrelates with time, representing the drift in the N-dimensional feature space. This pattern is consistent with the low-rank correction in Eq. **16**. The diffusion in z(t) in Eq. **16** is compensated for by a continuous alignment of $a_{t}$ to read out the target labels.

These results provide insights regarding the pattern of representational drift in neural circuits and the robustness of the performance to the drift. The results predict that injecting synaptic noise that changes the representation in a less constrained manner may result in a degraded performance, and can be tested with perturbation experiments. Finally, our recent work (39) has shown that architectural constraints such as the type of nonlinearity and weight sharing may result in significant performance in the presence of weight drift even in the absence of gradient error signal.

## Discussion

In this paper, we have pursued the hypothesis that geometric properties of neuronal representations offer natural order parameters for understanding the neuronal processing of high cognitive functions, and for comparing learning and computations in brains and in artificial neural networks. Recent work (65) suggested that long training times in DNNs trained for classification tasks result in “neural collapse”, where the feature layer representations of each class collapse to a single “prototype” point, hence the geometry of the intra-class (i.e., manifold) variability becomes irrelevant. However, empirical inspection of feature layer representations in DNNs and IT cortex does not support the “neural collapse” hypothesis (10, 11, 66). Most current work on manifold geometry focused on representation of discrete categories. This raises a host of open questions: How are relationships between multiple objects in the visual scene represented in neural activity? How does context shape the representation? And can we develop similar geometric theories to understand how brains and DNNs process language? Recent works (67, 68) have shown a surprising similarity between language processing in the human brain and large language models, raising fundamental questions about machine and human cognition and intelligence. Extending the theory of normative and mechanistic principles underlying language processing will make an important contribution to the field.

Our theoretical framework for learning in DNNs opens up several interesting future directions: Computing the theoretical predictor statistics requires inverting the P×P (renormalized) data kernel matrix $\tilde{K}$. Developing efficient algorithms for inverting large kernel matrices is an active research direction (69). Incorporating these methods into our framework will allow us to generate theoretical predictions on larger datasets in more realistic settings.

Applying our BPKR results to nonlinear networks is based on a heuristic ansatz, and works surprisingly well in certain parameter regime (Fig. 5*B*). Empirically it breaks down when L becomes large or in a single layer case when P is of the order of $N\cdot N_{0}$ (38). Theoretical effort to justify the approximation and delineate the regime where it is expected to fail is an important ongoing research direction (53, 54). This challenge motivated us to study a family of nonlinear deep networks, the globally gated deep linear network (37), in which a fully trained linear DNN architecture interacts multiplicatively with pre-trained or random nonlinear gated units. Gating networks have received attention recently both for their computational properties and for their relevance to multiplicative gating interactions in biological circuits (70, 71). We have shown that in these gated linear networks the renormalization effect is not canceled in the mean predictor as in the fully-connected DNNs. Indeed we have shown examples where the renormalization improves generalization through modifying the mean predictor. Previous works focusing on finite width corrections to the NNGP theory for CNNs have shown interesting results of feature learning (72, 73). Recent work applied the BPKR approach to CNNs with one hidden layer and likewise showed that the kernel undergoes local renormalization (74). Extending the theory to deep CNNs may better explain the emergence of observed patterns of representational geometry.

Another crucial choice concerns scaling the predictor with the network width. We have primarily focused on the lazy regime. Recent studies indicate that nonlazy scaling results in more pronounced feature learning (35, 36). This observation aligns with our findings from Eq. **15** that the feature learning component in the lazy regime becomes increasingly dominant when the variance of the prior weights $\sigma^{2}$ decreases. Deriving a theory of BNNs in the non-lazy regime is an important challenge.

Examining DNNs as models of brain cognitive systems has advanced computational and cognitive neuroscience. As our analysis in Fig. 3 demonstrates, such studies have the potential to reveal not only similarities but also important differences between current DNNs and the brain. Understanding these similarities as well as the discrepancies and their functional consequences should be on the agenda of theoretical neuroscience in the age of AI.

## Supplementary Material

Appendix 01 (PDF)

## Data Availability

Previously published data were used for this work (15, 57).

## References

[1] A. Zador , Catalyzing next-generation artificial intelligence through neuroAI. Nat. Commun. **14**, 1597 (2023). 36949048 10.1038/s41467-023-37180-x PMC10033876

[2] T. J. Sejnowski, The unreasonable effectiveness of deep learning in artificial intelligence. Proc. Natl. Acad. Sci. U.S.A. **117**, 30033–30038 (2020). 31992643 10.1073/pnas.1907373117 PMC7720171

[3] C. Tan ., “A survey on deep transfer learning” in *Artificial Neural Networks and Machine Learning–ICANN 2018: 27th International Conference on Artificial Neural Networks, Rhodes, Greece, October 4–7, 2018, Proceedings, Part III 27*, N. Lawrence, Eds. (Springer, 2018), pp. 270–279.

[4] Y. Wang, Q. Yao, J. T. Kwok, L. M. Ni, Generalizing from a few examples: A survey on few-shot learning. ACM Comp. Surv. (CSUR) **53**, 1–34 (2020).

[5] S. Bernardi , The geometry of abstraction in the hippocampus and prefrontal cortex. Cell **183**, 954–967 (2020). 33058757 10.1016/j.cell.2020.09.031 PMC8451959

[6] R. Chaudhuri, B. Gerçek, B. Pandey, A. Peyrache, I. Fiete, The intrinsic attractor manifold and population dynamics of a canonical cognitive circuit across waking and sleep. Nat. Neurosci. **22**, 1512–1520 (2019). 31406365 10.1038/s41593-019-0460-x

[7] R. J. Gardner , Toroidal topology of population activity in grid cells. Nature **602**, 123–128 (2022). 35022611 10.1038/s41586-021-04268-7 PMC8810387

[8] Y. Xie , Geometry of sequence working memory in macaque prefrontal cortex. Science **375**, 632–639 (2022). 35143322 10.1126/science.abm0204

[9] S. Chung, U. Cohen, H. Sompolinsky, D. D. Lee, Learning data manifolds with a cutting plane method. Neural Comput. **30**, 2593–2615 (2018). 30148702 10.1162/neco_a_01119

[10] U. Cohen, S. Y. Chung, D. D. Lee, H. Sompolinsky, Separability and geometry of object manifolds in deep neural networks. Nat. Commun. **11**, 1–13 (2020). 32029727 10.1038/s41467-020-14578-5 PMC7005295

[11] B. Sorscher, S. Ganguli, H. Sompolinsky, Neural representational geometry underlies few-shot concept learning. Proc. Natl. Acad. Sci. U.S.A. **119**, e2200800119 (2022). 36251997 10.1073/pnas.2200800119 PMC9618072

[12] J. J. DiCarlo, D. D. Cox, Untangling invariant object recognition. Trends Cognit. Sci. **11**, 333–341 (2007). 17631409 10.1016/j.tics.2007.06.010

[13] S. Chung, D. D. Lee, H. Sompolinsky, Classification and geometry of general perceptual manifolds. Phys. Rev. X **8**, 031003 (2018).

[14] A. J. Wakhloo, T. J. Sussman, S. Chung, Linear classification of neural manifolds with correlated variability. Phys. Rev. Lett. **131**, 027301 (2023). 37505944 10.1103/PhysRevLett.131.027301

[15] J. Deng ., “Imagenet: A large-scale hierarchical image database” in *2009 IEEE Conference on Computer Vision and Pattern Recognition* (IEEE, 2009), pp. 248–255.

[16] A. Krizhevsky, I. Sutskever, G. E. Hinton, Imagenet classification with deep convolutional neural networks. Adv. Neural Inf. Process. Syst. **25** (2012).

[17] K. Simonyan, A. Zisserman, Very deep convolutional networks for large-scale image recognition. arXiv [Preprint] (2014). http://arxiv.org/abs/1409.1556 (Accessed 28 November 2023).

[18] K. He, X. Zhang, S. Ren, J. Sun, “Deep residual learning for image recognition” in *Proceedings of the IEEE Conference on Computer Vision and Pattern Recognition* (2016), pp. 770–778.

[19] C. Cortes, V. Vapnik, Support-vector networks. Mach. Learn. **20**, 273–297 (1995).

[20] M. Schrimpf ., Brain-score: Which artificial neural network for object recognition is most brain-like? bioRxiv [Preprint] (2018). https://www.biorxiv.org/content/10.1101/407007v1 (Accessed 28 November 2023).

[21] N. J. Majaj, H. Hong, E. A. Solomon, J. J. DiCarlo, Simple learned weighted sums of inferior temporal neuronal firing rates accurately predict human core object recognition performance. J. Neurosci. **35**, 13402–13418 (2015). 26424887 10.1523/JNEUROSCI.5181-14.2015 PMC4588611

[22] P. Gao ., A theory of multineuronal dimensionality, dynamics and measurement. bioRxiv [Preprint] (2017). https://www.biorxiv.org/content/10.1101/214262v2 (Accessed 28 November 2023).

[23] A. Ansuini, A. Laio, J. H. Macke, D. Zoccolan, Intrinsic dimension of data representations in deep neural networks. Adv. Neural Inf. Process. Syst. **32** (2019).

[24] S. Recanatesi ., Dimensionality compression and expansion in deep neural networks. arXiv [Preprint] (2019). http://arxiv.org/abs/1906.00443 (Accessed 28 November 2023).

[25] D. Doimo, A. Glielmo, A. Ansuini, A. Laio, Hierarchical nucleation in deep neural networks. Adv. Neural Inf. Process. Syst. **33**, 7526–7536 (2020).

[26] L. Petrini, F. Cagnetta, U. M. Tomasini, A. Favero, M. Wyart, How deep neural networks learn compositional data: The random hierarchy model. arXiv [Preprint] (2023). http://arxiv.org/abs/2307.02129 (Accessed 28 November 2023).

[27] J. Lee ., Deep neural networks as Gaussian processes. arXiv [Preprint] (2017). http://arxiv.org/abs/1711.00165 (Accessed 28 November 2023).

[28] A. Jacot, F. Gabriel, C. Hongler, Neural tangent kernel: Convergence and generalization in neural networks. Adv. Neural Inf. Process. Syst. **31** (2018).

[29] A. Bietti, J. Mairal, On the inductive bias of neural tangent kernels. Adv. Neural Inf. Process. Syst. **32** (2019).

[30] S. Arora , On exact computation with an infinitely wide neural net. Adv. Neural Inf. process. Syst. **32** (2019).

[31] A. Canatar, B. Bordelon, C. Pehlevan, Spectral bias and task-model alignment explain generalization in kernel regression and infinitely wide neural networks. Nat. Commun. **12**, 2914 (2021). 34006842 10.1038/s41467-021-23103-1 PMC8131612

[32] L. Chizat, F. Bach, “Implicit bias of gradient descent for wide two-layer neural networks trained with the logistic loss” in *Conference on Learning Theory*, N. Lawrence, Eds. (PMLR, 2020), pp. 1305–1338.

[33] J. Lee , Wide neural networks of any depth evolve as linear models under gradient descent. Adv. Neural Inf. Process. Syst. **32** (2019).

[34] B. Bordelon, A. Canatar, C. Pehlevan, “Spectrum dependent learning curves in kernel regression and wide neural networks” in *International Conference on Machine Learning*, N. Lawrence, Eds. (PMLR, 2020), pp. 1024–1034.

[35] B. Bordelon, C. Pehlevan, The influence of learning rule on representation dynamics in wide neural networks. arXiv [Preprint] (2022). http://arxiv.org/abs/2210.02157 (Accessed 28 November 2023).

[36] B. Bordelon, C. Pehlevan, Self-consistent dynamical field theory of kernel evolution in wide neural networks. Adv. Neural Inf. Process. Syst. **35**, 32240–32256 (2022).

[37] Q. Li, H. Sompolinsky, Globally gated deep linear networks. Adv. Neural Inf. Process. Syst. **35**, 34789–34801 (2022).

[38] Q. Li, H. Sompolinsky, Statistical mechanics of deep linear neural networks: The backpropagating kernel renormalization. Phys. Rev. X **11**, 031059 (2021).

[39] Y. Avidan, Q. Li, H. Sompolinsky, Connecting NTK and NNGP: A unified theoretical framework for neural network learning dynamics in the kernel regime. arXiv [Preprint] (2023). http://arxiv.org/abs/2309.04522 (Accessed 28 November 2023).

[40] J. Larsen, L. K. Hansen, “Generalization performance of regularized neural network models” in *Proceedings of IEEE Workshop on Neural Networks for Signal Processing* (IEEE, Ermioni, Greece, 1994), pp. 42–51.

[41] A. Krogh, J. Hertz, A simple weight decay can improve generalization. Adv. Neural Inf. Process. Syst. **4** (1991).

[42] F. Mignacco, F. Krzakala, P. Urbani, L. Zdeborová, Dynamical mean-field theory for stochastic gradient descent in gaussian mixture classification. Adv. Neural Inf. Process. Syst. **33**, 9540–9550 (2020).

[43] C. Gerbelot, E. Troiani, F. Mignacco, F. Krzakala, L. Zdeborova, Rigorous dynamical mean field theory for stochastic gradient descent methods. arXiv [Preprint] (2022). http://arxiv.org/abs/2210.06591 (Accessed 28 November 2023).

[44] G. Ben Arous, R. Gheissari, A. Jagannath, High-dimensional limit theorems for SGD: Effective dynamics and critical scaling. Adv. Neural Inf. Process. Syst. **35**, 25349–25362 (2022).

[45] K. Segadlo , Unified field theoretical approach to deep and recurrent neuronal networks. J. Stat. Mech.: Theory Exp. **2022**, 103401 (2022).

[46] M. Geiger, S. Spigler, A. Jacot, M. Wyart, Disentangling feature and lazy training in deep neural networks. J. Stat. Mech.: Theory Exp. **2020**, 113301 (2020).

[47] J. Lee , Finite versus infinite neural networks: An empirical study. Adv. Neural Inf. Process. Syst. **33**, 15156–15172 (2020).

[48] R. M. Neal , MCMC using Hamiltonian dynamics. Handb. Markov Chain Monte Carlo **2**, 2 (2011).

[49] A. Vehtari, S. Sarkka, J. Lampinen, “On MCMC sampling in Bayesian MLP neural networks” in *Proceedings of the IEEE-INNS-ENNS International Joint Conference on Neural Networks. IJCNN 2000. Neural Computing: New Challenges and Perspectives for the New Millennium* (IEEE, Como, Italy, 2000), vol. 1, pp. 317–322.

[50] W. Coffey, Y. P. Kalmykov, The Langevin Equation: With Applications to Stochastic Problems in Physics, Chemistry and Electrical Engineering (World Scientific, 2012), vol. 27.

[51] M. Advani, S. Lahiri, S. Ganguli, Statistical mechanics of complex neural systems and high dimensional data. J. Stat. Mech.: Theory Exp. **2013**, P03014 (2013).

[52] B. Hanin, A. Zlokapa, Bayesian interpolation with deep linear networks. Proc. Natl. Acad. Sci. U.S.A. **120**, e2301345120 (2023). 37252994 10.1073/pnas.2301345120 PMC10266010

[53] S. Ariosto ., Statistical mechanics of deep learning beyond the infinite-width limit. arXiv [Preprint] (2022). http://arxiv.org/abs/2209.04882 (Accessed 28 November 2023).

[54] H. Cui, F. Krzakala, L. Zdeborová, Optimal learning of deep random networks of extensive-width. arXiv [Preprint] (2023). http://arxiv.org/abs/2302.00375 (Accessed 28 November 2023).

[55] H. Hu, Y. M. Lu, Universality laws for high-dimensional learning with random features. IEEE Trans. Inf. Theory **69**, 1932–1964 (2022).

[56] S. Dubova, Y. M. Lu, B. McKenna, H. T. Yau, Universality for the global spectrum of random inner-product kernel matrices in the polynomial regime. arXiv [Preprint] (2023). http://arxiv.org/abs/2310.18280 (Accessed 28 November 2023).

[57] L. Deng, The MNIST database of handwritten digit images for machine learning research. IEEE Sig. Process. Magaz. **29**, 141–142 (2012).

[58] M. E. Rule, T. O’Leary, C. D. Harvey, Causes and consequences of representational drift. Curr. Opin. Neurobiol. **58**, 141–147 (2019). 31569062 10.1016/j.conb.2019.08.005 PMC7385530

[59] S. Druckmann, D. B. Chklovskii, Neuronal circuits underlying persistent representations despite time varying activity. Curr. Biol. **22**, 2095–2103 (2012). 23084992 10.1016/j.cub.2012.08.058 PMC3543774

[60] M. T. Kaufman, M. M. Churchland, S. I. Ryu, K. V. Shenoy, Cortical activity in the null space: Permitting preparation without movement. Nat. Neurosci. **17**, 440–448 (2014). 24487233 10.1038/nn.3643 PMC3955357

[61] A. Rubin , Revealing neural correlates of behavior without behavioral measurements. Nat. Commun. **10**, 4745 (2019). 31628322 10.1038/s41467-019-12724-2 PMC6802184

[62] D. Deitch, A. Rubin, Y. Ziv, Representational drift in the mouse visual cortex. Curr. Biol. **31**, 4327–4339 (2021). 34433077 10.1016/j.cub.2021.07.062

[63] M. E. Rule , Stable task information from an unstable neural population. Elife **9**, e51121 (2020). 32660692 10.7554/eLife.51121 PMC7392606

[64] F. Pashakhanloo, A. Koulakov, Stochastic gradient descent-induced drift of representation in a two-layer neural network. arXiv [Preprint] (2023). http://arxiv.org/abs/2302.02563 (Accessed 28 November 2023).

[65] V. Papyan, X. Han, D. L. Donoho, Prevalence of neural collapse during the terminal phase of deep learning training. Proc. Natl. Acad. Sci. U.S.A. **117**, 24652–24663 (2020). 32958680 10.1073/pnas.2015509117 PMC7547234

[66] L. Hui, M. Belkin, P. Nakkiran, Limitations of neural collapse for understanding generalization in deep learning. arXiv [Preprint] (2022). http://arxiv.org/abs/2202.08384 (Accessed 28 November 2023).

[67] J. Li ., Large language models converge on brain-like word representations. arXiv [Preprint] (2023). http://arxiv.org/abs/2306.01930 (Accessed 28 November 2023).

[68] A. Goldstein ., Correspondence between the layered structure of deep language models and temporal structure of natural language processing in the human brain. bioRxiv [Preprint] (2022). https://www.biorxiv.org/content/10.1101/2022.07.11.499562v2 (Accessed 28 November 2023).

[69] R. Novak, J. Sohl-Dickstein, S. S. Schoenholz, “Fast finite width neural tangent kernel” in *International Conference on Machine Learning*, N. Lawrence, Eds. (PMLR, 2022), pp. 17018–17044.

[70] E. Sezener ., A rapid and efficient learning rule for biological neural circuits. BioRxiv [Preprint] (2021). https://www.biorxiv.org/content/10.1101/2021.03.10.434756v1 (Accessed 28 November 2023).

[71] A. Saxe, S. Sodhani, S. J. Lewallen, “The neural race reduction: Dynamics of abstraction in gated networks” in *International Conference on Machine Learning*, N. Lawrence, Eds. (PMLR, 2022), pp. 19287–19309.

[72] G. Naveh, Z. Ringel, A self consistent theory of Gaussian processes captures feature learning effects in finite CNNs. Adv. Neural Inf. Process. Syst. **34**, 21352–21364 (2021).

[73] I. Seroussi, G. Naveh, Z. Ringel, Separation of scales and a thermodynamic description of feature learning in some CNNs. Nat. Commun. **14**, 908 (2023). 36804926 10.1038/s41467-023-36361-y PMC9938275

[74] R. Aiudi, R. Pacelli, A. Vezzani, R. Burioni, P. Rotondo, Local kernel renormalization as a mechanism for feature learning in overparametrized convolutional neural networks. arXiv [Preprint] (2023). http://arxiv.org/abs/2307.11807 (Accessed 28 November 2023). 10.1038/s41467-024-55229-3 PMC11724055 39794337

[75] J. Hron, Y. Bahri, J. Sohl-Dickstein, R. Novak, “Infinite attention: NNGP and NTK for deep attention networks” in *International Conference on Machine Learning*, A. Singh, H. Daume, Eds. (JMLR.org, PMLR, Ermioni, Greece, 2020), pp. 4376–4386.

