# Supplementary material for: Representations and generalization in artificial and brain neural networks
Source: Proc Natl Acad Sci U S A. 2024 Jun 24;121(27):e2311805121. doi: 10.1073/pnas.2311805121 (PMC11228472; doi:10.1073/pnas.2311805121)
Supplement: Supplementary file 1 — Appendix 01 (PDF) [file pnas.2311805121.sapp.pdf]

Supporting Information for

Representations and Generalization in Artificial and Brain Neural Networks

Qianyi Li, Ben Sorscher and Haim Sompolinsky

Haim Sompolinsky.
E-mail: hsompolinsky@mcb.harvard.edu

This PDF file includes:

Supporting text
SI References

Contents

1 Detailed derivation of new theoretical results in the "Theory of Deep Learning" section **2**

A Mean layer-wise kernels . . . 2

B From mean layer-wise kernel to geometry . . . 3

2 Details of new numerical simulations in Fig.6-8 **4**

Supporting Information Text

1. Detailed derivation of new theoretical results in the "Theory of Deep Learning" section

A. Mean layer-wise kernels. In (1), we derived the mean layer-wise kernel matrices on the training data, but not the mean layer-wise kernel function. Here we start from Eq.D7 and Eq.C10 in (1) and provide detailed derivation of the general expression of $K_l(\mathbf{x},\mathbf{x}')$ applied on arbitrary points $\{\mathbf{x},\mathbf{x}'\}$ for deep linear networks, and propose our ansatz for nonlinear DNNs. From Eq.D7 in (1), we have for linear DNNs

$$\langle W^l {W^l}^\top \rangle_{W^l} = \sigma^2 I - \frac{\sigma^4}{N^2}[M\phi(h^{l-1}(\mathbf{X}))^\top \mathbf{K}_{l-1}^{-1}\phi(h^{l-1}(\mathbf{X})) - \phi(h^{l-1}(\mathbf{X}))^\top \mathbf{K}_{l-1}^{-1}\mathbf{Y}\mathcal{U}_{l-1}^{-(L-l+1)}\mathbf{Y}^\top \mathbf{K}_{l-1}^{-1}\phi(h^{l-1}(\mathbf{X}))] \quad [1]$$

Here $\langle\rangle_{W^l}$ denotes partial averaging over the conditional distribution

$$P(\mathbf{a}, W^L, \cdots, W^l | W^{l-1}, \cdots, W^1) \quad [2]$$

Therefore the RHS of Eq.1 still depends on the remaining weights $W^{l-1}\cdots W^1$. $\mathbf{K}_l$ denotes the $\mathcal{W}$-dependent $P\times P$ data kernel matrix as defined in the main text, and $h^{l-1}(\mathbf{X})\in\mathbb{R}^{P\times N}$ denotes the $\mathcal{W}$-dependent hidden layer pre-activation on the training data $\mathbf{X}$. $\mathcal{U}_{l-1}$ denotes the renormalization factor introduced during partial integration, and depends on the remaining weights through the saddle-point equation (Eq.34 in (1))

$$\mathbb{I} - \left(\frac{\mathcal{U}_{l-1}}{\sigma^2}\right) = \alpha\left(\mathbb{I} - \left(\frac{\mathcal{U}_{l-1}}{\sigma^2}\right)^{-(L-l+1)}\mathcal{R}_{l-1}\right); \mathcal{R}_{l-1} = \frac{1}{P}\mathbf{Y}^\top {\mathbf{K}_{l-1}^{GP}}^{-1}\mathbf{Y} \quad [3]$$

For linear networks, the conditional average of the layer-wise kernel is given by multiplying Eq.1 with $\phi(h^{l-1}(\mathbf{x}))$ and $\phi(h^{l-1}(\mathbf{x}'))$ on both sides, and scaled by $\frac{\sigma^2}{N}$, so we have

$$\langle K_l(\mathbf{x},\mathbf{x}')\rangle_{W_l} = \sigma^2 K_{l-1}(\mathbf{x},\mathbf{x}') - \frac{\sigma^2}{N}[M\mathbf{k}_{l-1}(\mathbf{x})^\top \mathbf{K}_{l-1}^{-1}\mathbf{k}_{l-1}(\mathbf{x}') - \mathbf{k}_{l-1}(\mathbf{x})^\top \mathbf{K}_{l-1}^{-1}\mathbf{Y}\mathcal{U}_{l-1}^{-(L-l+1)}\mathbf{Y}^\top \mathbf{K}_{l-1}^{-1}\mathbf{k}_{l-1}(\mathbf{x}')] \quad [4]$$

To average over the remaining weights, we need to average the last two terms in Eq.4, which is highly non-trivial as $\mathcal{W}$ appears in $\mathbf{k}_{l-1}(\mathbf{x})$, the inverse kernel $\mathbf{K}_{l-1}^{-1}$, and in the expression of $\mathcal{U}_{l-1}$. However, we notice that the last two terms can be related to the variance and mean of the predictor, conditioned on the remaining weights $W^1,\cdots,W^{l-1}$, which simplifies the calculation. To see this, we take Eq.C10 from (1), the moment-generating function (MGF) for the predictor statistics conditioned on $W^1,\cdots,W^{l-1}$, and we have

$$\begin{aligned}
Z_{l-1}(\ell,\mathbf{x}) &= \int d\mathcal{U}_{l-1}\int dt \exp\Big[i\mathrm{Tr}(t^\top\mathbf{Y}) + \frac{(N-P)(L-l+1)}{2}\log\det\mathcal{U}_{l-1} - \frac{N(L-l+1)}{2\sigma^2}\mathrm{Tr}(\mathcal{U}_{l-1}) - \frac{1}{2}\mathrm{Tr}(\mathcal{U}_{l-1}^{L-l+1}t^\top\mathbf{K}_{l-1}t) \\
&- \frac{1}{2\sigma^2}\sum_{l'=1}^{l-1}\mathrm{Tr}({W^{l'}}^\top W^{l'}) + \mathrm{Tr}(\mathcal{U}_{l-1}^{L-l+1}\ell^\top\mathbf{k}_{l-1}(\mathbf{x})^\top t) - \frac{1}{2}\mathrm{Tr}(\mathcal{U}_{l-1}^{L-l+1}\ell^\top K_{l-1}(\mathbf{x},\mathbf{x})\ell)\Big] \\
&= \int d\mathcal{U}_{l-1}\exp\Big\{\frac{(N-P)(L-l+1)}{2}\log\det\mathcal{U}_{l-1} - \frac{N(L-l+1)}{2\sigma^2}\mathrm{Tr}(\mathcal{U}_{l-1}) - \frac{1}{2}\mathrm{Tr}(\mathcal{U}_{l-1}^{L-l+1}\ell^\top K_{l-1}(\mathbf{x},\mathbf{x})\ell) - \frac{1}{2\sigma^2}\sum_{l'=1}^{l-1}\mathrm{Tr}({\mathcal{W}^{l'}}^\top\mathcal{W}^{l'}) \\
&+ \frac{1}{2}\mathrm{Tr}\left[\left(i\mathbf{K}_{l-1}^{-1/2}\mathbf{Y} + \mathbf{K}_{l-1}^{-1/2}\mathbf{k}_{l-1}(\mathbf{x})\ell^\top\mathcal{U}_{l-1}^{L-l+1}\right)\mathcal{U}_{l-1}^{-(L-l+1)}\left(i\mathbf{K}_{l-1}^{-1/2}\mathbf{Y} + \mathbf{K}_{l-1}^{-1/2}\mathbf{k}_{l-1}(\mathbf{x})\ell^\top\mathcal{U}_{l-1}^{L-l+1}\right)^\top\right]\Big\}
\end{aligned} \quad [5]$$

where t is an auxilliary integration variable which we integrate out in the second equality. ℓ is the source coupled to the predictor $f(\mathbf{x})$ in the MGF. Taking derivatives w.r.t. ℓ, we obtain the partially averaged predictor statistics.

$$\begin{aligned}
\langle f(\mathbf{x})\rangle_{W^l} &= \mathbf{k}_{l-1}(\mathbf{x})^\top\mathbf{K}_{l-1}^{-1}\mathbf{Y} \\
\langle \delta f(\mathbf{x})^\top\delta f(\mathbf{x}')\rangle_{W^l} &= \mathcal{U}_{l-1}^{L-l+1}\left(K_{l-1}(\mathbf{x},\mathbf{x}') - \mathbf{k}_{l-1}(\mathbf{x})^\top\mathbf{K}_{l-1}^{-1}\mathbf{k}_{l-1}(\mathbf{x}')\right)
\end{aligned} \quad [6]$$

Comparing the last two terms in Eq.4 with Eq.6, we see that they can be rewritten as

$$\begin{aligned} & M\mathbf{k}_{l-1}(\mathbf{x})^\top \mathbf{K}_{l-1}^{-1}\mathbf{k}_{l-1}(\mathbf{x}') - \mathbf{k}_{l-1}(\mathbf{x})^\top \mathbf{K}_{l-1}^{-1}\mathbf{Y}\mathcal{U}_{l-1}^{-(L-l+1)}\mathbf{Y}^\top \mathbf{K}_{l-1}^{-1}\mathbf{k}_{l-1}(\mathbf{x}') \\ =& MK_{l-1}(\mathbf{x},\mathbf{x}') - \mathrm{Tr}[\mathcal{U}_{l-1}^{-(L-l+1)}(\langle \delta f(\mathbf{x})^\top \delta f(\mathbf{x}')\rangle_{W^l} + \langle f(\mathbf{x})\rangle_{W^l}^\top \langle f(\mathbf{x})\rangle_{W^l})] \\ =& MK_{l-1}(\mathbf{x},\mathbf{x}') - \mathrm{Tr}[\mathcal{U}_{l-1}^{-(L-l+1)}\langle f(\mathbf{x})^\top f(\mathbf{x}')\rangle_{W^l}] \end{aligned} \quad [7]$$

Therefore, Eq.4 is equivalent to

$$\langle K_l(\mathbf{x},\mathbf{x}')\rangle_{W^l} = \sigma^2(1-\frac{M}{N})K_{l-1}(\mathbf{x},\mathbf{x}') + \mathrm{Tr}[\mathcal{U}_{l-1}^{-(L-l+1)}\langle f(\mathbf{x})^\top f(\mathbf{x}')\rangle_{W^l}] \quad [8]$$

To average over the remaining weights, we only need to evaluate $\langle K_{l-1}(\mathbf{x},\mathbf{x}')\rangle_\Theta$, $\langle \mathcal{U}_{l-1}^{-(L-l+1)}\rangle_\Theta$, and $\langle f(\mathbf{x})f(\mathbf{x})^\top\rangle_\Theta$ (as in (1), we always average the weights first, then calculate saddle point approximation on the renormalization factors, therefore the average over $\mathcal{U}_{l-1}^{-(L-l+1)}$ and $\langle f(\mathbf{x})f(\mathbf{x})^\top\rangle_\Theta$ in the second term of Eq.8 can be performed separately). Using Eq.C9 in (1), we have

$$\langle K_l(\mathbf{x},\mathbf{x}')\rangle_\Theta = \sigma^2(1-\frac{M}{N})\langle K_{l-1}(\mathbf{x},\mathbf{x}')\rangle_\Theta + \mathrm{Tr}[\mathcal{U}_0^{-(L-l+1)}\langle f(\mathbf{x})^\top f(\mathbf{x}')\rangle_\Theta] \quad [9]$$

Iterating this recursive relation and using the expression for the predictor statistics,

$$\langle f(\mathbf{x})^\top f(\mathbf{x}')\rangle_\Theta = \mathcal{U}_0^L\sigma^{2L}[K_0(\mathbf{x},\mathbf{x}') - \mathbf{k}_0(\mathbf{x})^\top \mathbf{K}_0^{-1}\mathbf{k}_0(\mathbf{x}')] + \mathbf{Y}^\top \mathbf{K}_0^{-1}\mathbf{k}_0(\mathbf{x}')\mathbf{k}_0(\mathbf{x})^\top \mathbf{K}_0^{-1}\mathbf{Y} \quad [10]$$

where $K_0(\mathbf{x},\mathbf{x}')$ is the input kernel function given by $K_0(\mathbf{x},\mathbf{x}') = \frac{\sigma^2}{N}\mathbf{x}\cdot\mathbf{x}'$, and $\mathbf{k}_0(\mathbf{x}) \in \mathbb{R}^P$ and $\mathbf{K}_0 \in \mathbb{R}^{P\times P}$ are defined correspondingly as K_0 applied on testing and training data, we have

$$\begin{aligned} \langle K_l(\mathbf{x},\mathbf{x}')\rangle_\Theta =& \sigma^{2l}(1-\frac{M}{N})^l K_0(\mathbf{x},\mathbf{x}') + \frac{\sigma^{2l}}{N}\mathrm{Tr}\left[\frac{\mathbb{I}-\sigma^{-2l}\mathcal{U}_0^l}{\mathbb{I}-\sigma^{-2}\mathcal{U}_0}\right]\cdot[K_0(\mathbf{x},\mathbf{x}') - \mathbf{k}_0(\mathbf{x})^\top \mathbf{K}_0^{-1}\mathbf{k}_0(\mathbf{x}')] \\ &+ \frac{\sigma^{2(l-1)}}{N}\mathbf{k}_0(\mathbf{x})^\top \mathbf{K}_0^{-1}\mathbf{Y}\frac{\mathbb{I}-\sigma^{-2l}\mathcal{U}_0^l}{\mathcal{U}_0^L(\mathbb{I}-\sigma^{-2}\mathcal{U}_0)}\mathbf{Y}^\top \mathbf{K}_0^{-1}\mathbf{k}_0(\mathbf{x}) \end{aligned} \quad [11]$$

For general nonlinear networks, we propose a similar ansatz as in (1), where we replace $\sigma^{2l}K_0$ with the corresponding NNGP kernel K_l^{GP}. Denoting the NNGP predictor statistics of an l-layer network as

$$\begin{aligned} \langle f_l(\mathbf{x})\rangle &= \mathbf{k}_l^{GP}(\mathbf{x})^\top \mathbf{K}_l^{GP\,-1}\mathbf{Y} \\ \langle \delta f_l(\mathbf{x})^\top \delta f_l(\mathbf{x}')\rangle &= \mathbb{I}_M\cdot(K_l^{GP}(\mathbf{x},\mathbf{x}') - \mathbf{k}_l^{GP}(\mathbf{x})^\top \mathbf{K}_l^{GP\,-1}\mathbf{k}_l^{GP}(\mathbf{x}')) \end{aligned} \quad [12]$$

and define function of $\mathcal{U}_0$, $G_l(\mathcal{U}_0) = \frac{\sigma^{2(l-1)}\mathbb{I}-\sigma^{-2}\mathcal{U}_0^l}{\mathcal{U}_0^L(\mathbb{I}-\sigma^{-2}\mathcal{U}_0)}$, we have

$$\langle K_l(\mathbf{x},\mathbf{x}')\rangle_\Theta = (1-\frac{M}{N})^l K_l^{GP}(\mathbf{x},\mathbf{x}') + \frac{1}{N}\mathrm{Tr}[\sigma^{-2(l-1)}\mathcal{U}_0^{-L}G_l(\mathcal{U}_0)\langle \delta f_l(\mathbf{x})^\top \delta f_l(\mathbf{x}')\rangle] + \frac{1}{N}\langle f_l(\mathbf{x})\rangle G_l(\mathcal{U}_0)\langle f(\mathbf{x}')\rangle^\top \quad [13]$$

On the training data, the NNGP predictor variance goes to zero, and the mean predictor is the target training labels $\mathbf{Y}$. Therefore, the second term vanishes, and $\langle f(\mathbf{x})\rangle_\Theta = \mathbf{Y}$for the training data kernel matrix. Therefore we have

$$\langle \mathbf{K}_l\rangle_\Theta = (1-\frac{M}{N})^l \mathbf{K}_l^{GP} + \frac{1}{N}\mathbf{Y}\rangle G_l(\mathcal{U}_0)\mathbf{Y}^\top \quad [14]$$

In the main text, for simplicity, we neglect the $(1-\frac{M}{N})^l$ factor in front of $K_l^{GP}(\mathbf{x},\mathbf{x}')$ as it is always small for large N, irrespective of σ. We do take this term into account in the numerical results we present.

B. From mean layer-wise kernel to geometry. In this section we provide detailed derivations and expressions of the geometric metrics $\|\Delta\mathbf{x}_0\|^2$ and D calculated from the mean layer-wise kernel function.

Consider two manifolds a and b, and a set of data points $\mathbf{x}_a^1,\cdots,\mathbf{x}_a^{P_a}$ and $\mathbf{x}_b^1,\cdots,\mathbf{x}_b^{P_b}$, from the two manifolds, respectively. The geometric properties as defined in the main text are functions of the representations of these data points in the hidden layers, given by $\{\phi(h^l(\mathbf{x}_a^\mu))\}_{\mu=1,\cdots P_a}$, $\{\phi(h^l(\mathbf{x}_b^\nu))\}_{\nu=1,\cdots P_b}$. Specifically, the signal $\|\Delta\mathbf{x}_0\|^2$ and the dimension D can be evaluated from the inner product of the hidden layer activations, which is equivalent to the $\mathcal{W}$-dependent kernels as defined in the main text,

$$K_l(\mathbf{x}_m^\mu,\mathbf{x}_n^\nu) = \frac{\sigma^2}{N}\phi(h^l(\mathbf{x}_m^\mu))\cdot\phi(h^l(\mathbf{x}_n^\nu)); m,n\in\{a,b\}; \mu=1,\cdots,P_m; \nu=1,\cdots,P_n \quad [15]$$

We show below the detailed expressions.

1. **Radius**. As defined in the main text, the squared radius R_a^2 measures the overall size of the variation of the manifold around its center. By definition, it is equivalent to the normalized trace of the covariance matrix of the corresponding manifold. We can evaluate the $N \times N$ empirical covariance matrix by

$$C = \frac{1}{P_a - 1} \sum_{\mu=1}^{P_a} (\phi(h^l(\mathbf{x}_a^\mu)) - \overline{\phi(h^l(\mathbf{x}_a))})(\phi(h^l(\mathbf{x}_a^\mu)) - \overline{\phi(h^l(\mathbf{x}_a))})^\top \quad [16]$$

where $\overline{\phi(h^l(\mathbf{x}_a))}$ denotes the average across all the data points within manifold a, $\overline{\phi(h^l(\mathbf{x}_a))} = \frac{1}{P_a} \sum_{\mu=1}^{P_a} \phi(h^l(\mathbf{x}_a^\mu))$. The squared radius is therefore given by

$$\begin{aligned} R_a^2 &= \frac{1}{N}\mathrm{Tr}(C) = \frac{1}{N(P_a-1)} \sum_{\mu=1}^{P_a} (\phi(h^l(\mathbf{x}_a^\mu)) - \overline{\phi(h^l(\mathbf{x}_a))}) \cdot (\phi(h^l(\mathbf{x}_a^\mu)) - \overline{\phi(h^l(\mathbf{x}_a))}) \\ &= \sigma^{-2}[\frac{1}{P_a-1} \sum_{\mu=1}^{P_a} K_l(\mathbf{x}_a^\mu, \mathbf{x}_a^\mu) - \frac{1}{P_a(P_a-1)} \sum_{\mu,\nu=1}^{P_a} K_l(\mathbf{x}_a^\mu, \mathbf{x}_a^\nu)] \end{aligned} \quad [17]$$

2. **Signal**. The signal between a pair of manifolds is defined as $\|\Delta\mathbf{x}_0\|^2 = \|\overline{\phi(h^l(\mathbf{x}_a))} - \overline{\phi(h^l(\mathbf{x}_b))}\|^2 / R_a^2$. The numerator is given by

$$\|\overline{\phi(h^l(\mathbf{x}_a))} - \overline{\phi(h^l(\mathbf{x}_b))}\|^2 = \sigma^{-2}[\frac{1}{P_a^2} \sum_{\mu,\nu=1}^{P_a} K_l(\mathbf{x}_a^\mu, \mathbf{x}_a^\nu) - \frac{2}{P_a P_b} \sum_{\mu=1}^{P_a} \sum_{\nu=1}^{P_b} K_l(\mathbf{x}_a^\mu, \mathbf{x}_b^\nu) + \frac{1}{P_b^2} \sum_{\mu,\nu=1}^{P_b} K_l(\mathbf{x}_b^\mu, \mathbf{x}_b^\nu)] \quad [18]$$

Combined with the radius we have

$$\|\Delta\mathbf{x}_0\|^2 = (R_a^2)^{-1} \cdot [\frac{1}{P_a^2} \sum_{\mu,\nu=1}^{P_a} K_l(\mathbf{x}_a^\mu, \mathbf{x}_a^\nu) - \frac{2}{P_a P_b} \sum_{\mu=1}^{P_a} \sum_{\nu=1}^{P_b} K_l(\mathbf{x}_a^\mu, \mathbf{x}_b^\nu) + \frac{1}{P_b^2} \sum_{\mu,\nu=1}^{P_b} K_l(\mathbf{x}_b^\mu, \mathbf{x}_b^\nu)] \quad [19]$$

3. **Dimension**. The dimension of a manifold a as defined in the main text can be evaluated by $\frac{(\mathrm{Tr}(C))^2}{\mathrm{Tr}(C^2)}$. The numerator is already given by Eq.17, the denominator can be given by

$$\begin{aligned} \mathrm{Tr}(C^2) &= \frac{1}{(P_a-1)^2} \sum_{\mu,\nu=1}^{P_a} [(\phi(h^l(\mathbf{x}_a^\mu)) - \overline{\phi(h^l(\mathbf{x}_a))}) \cdot (\phi(h^l(\mathbf{x}_a^\nu)) - \overline{\phi(h^l(\mathbf{x}_a))})]^2 \\ &= \frac{N^2\sigma^{-4}}{(P_a-1)^2} \sum_{\mu,\nu=1}^{P_a} [K_l(\mathbf{x}_a^\mu, \mathbf{x}_a^\nu) - \frac{1}{P_a} \sum_{\mu'=1}^{P_a} K_l(\mathbf{x}_a^\mu, \mathbf{x}_a^{\mu'}) - \frac{1}{P_a} \sum_{\nu'=1}^{P_a} K_l(\mathbf{x}_a^{\nu'}, \mathbf{x}_a^\nu) + \frac{1}{P_a^2} \sum_{\mu',\nu'=1}^{P_a} K_l(\mathbf{x}_a^{\mu'}, \mathbf{x}_a^{\nu'})]^2 \end{aligned} \quad [20]$$

Combined with the radius we have

$$D = (R_a^2)^2 N^2 [\sum_{\mu,\nu=1}^{P_a} [K_l(\mathbf{x}_a^\mu, \mathbf{x}_a^\nu) - \frac{1}{P_a} \sum_{\mu'=1}^{P_a} K_l(\mathbf{x}_a^\mu, \mathbf{x}_a^{\mu'}) - \frac{1}{P} \sum_{\nu'=1}^{P_a} K_l(\mathbf{x}_a^{\nu'}, \mathbf{x}_a^\nu) + \frac{1}{P_a^2} \sum_{\mu',\nu'=1}^{P_a} K_l(\mathbf{x}_a^{\mu'}, \mathbf{x}_a^{\nu'})]^2]^{-1} \quad [21]$$

To apply our theory for the mean layer-wise kernel, we replace K_l with its mean K_l in the above expressions in our preliminary evaluations shown in Fig.7 in the main text. We show the average D and $\|\Delta\mathbf{x}_0\|^2$ across all 10 manifolds and 90 pairs of manifolds in MNIST. More careful analysis and justifications of this approximation are ongoing work.

2. Details of new numerical simulations in Fig.6-8

Figure 6: (**A**-**B**) The NNGP data kernel matrix and the theoretical mean layer-wise data kernel matrix, for a 1-hidden-layer ReLU network with 7 outputs trained on classification of 8 MNIST digits (0-7). We properly normalize and center the input data such that it has zero mean and standard deviation 1. The output labels are 7-dimensional one-hot vectors encoding identity of the digits, and centered to have zero mean (so we only have 7 outputs and 8 classes, otherwise the outputs become linearly dependent). The parameters are $N_0 = 784$, $P = 1000$, $P_{test} = 1000$ (the number of test data point for evaluating the generalization error), $\alpha = 2.7$, $\sigma = 0.2$.
(**C**) Data and all the parameters are the same as in (**A**-**B**), except α is varied through changing N. For the random feature model, we consider a network with the same architecture but standard Gaussian i.i.d. $\mathcal{W}$, generalization error is calculated by averaging over 5000 different samples of $\mathcal{W}$.
(**D**) A 4-hidden-layer ReLU network trained on a subset of MNIST data with four different digits (2,3,4,5). The output of the network is six dimensional ($y \in \{0, 1\}^6$), designed to have hierarchical block structure; four of the binary outputs are one-hot vectors each encoding one digit. The four digits are divided into two categories (even vs. odd); the other two binary outputs

each classify one of the two categories. The parameters are $N_0 = 784$, $P = 100$, $P_{test} = 1000$, $\alpha = 0.1$, $\sigma = 0.1$.

Figure 7: (**A**-**B**) Signal and dimension computed from the mean layer-wise kernel on test data points, for a 7-hidden-layer ReLU network and a linear network of the same architecture trained to perform 10-way classification on MNIST. The input data is properly centered and normalized to have zero mean and standard deviation 1. The output labels are 10-dimensional one-hot vectors encoding identity of the digits. The parameters are $N_0 = 784$, $P = 1000$, $P_{test} = 1000$, $\alpha = 0.2$, $\sigma = 0.8$.

Figure 8: (**A**-**B**) Langevin learning dynamics of a single layer ReLU network trained on binary MNIST classification of digits 0 and 1. The input data is properly normalized to have zero mean and standard deviation 1. The output is ± 1. For the blue curve, to demonstrate the degraded learning performance without the presence of a learning signal during the drift, $\mathbf{a}$ is fixed at $t = 100$ where the training error is sufficiently small, and $\mathcal{W}$ drifts randomly sampling from the Gaussian prior afterwards. In (**B**), we track the dynamics of the hidden layer activation $\phi(h_t^l(\mathbf{X})) \in \mathbb{R}^{N \times P}$ by looking at its top left and right singular vectors. Since the mean value of the activation may potentially contribute to a large singular value, we subtract the corresponding mean before evaluating $\mathbf{u}(t)$ and $\mathbf{v}(t)$. The parameters are $N_0 = 784$, $P = 100$, $P_{test} = 1500$, $\alpha = 0.2$, $\sigma = 0.2$, $dt = 0.01$ (learning rate), $t_0 = 100$ ($t_0 = dt \times (number\ of\ steps)$), $\beta = 1e4$.

References

1. Q Li, H Sompolinsky, Statistical mechanics of deep linear neural networks: The backpropagating kernel renormalization. *Phys. Rev. X* **11**, 031059 (2021).
